# Radiolysis study on PVDF binder – how does it affect the X-ray photoelectron spectroscopy characterisation of phospho-olivine electrodes?

**DOI:** 10.1039/d6eb00103c

**Published:** 2026-08-07

**Authors:** Yuhan Liu, Prajna Bhatt, Will J. Dawson, Alice Llewellyn, Joshua Dickerson, Robert Palgrave, Anna Regoutz, James Robinson, Rhodri Jervis

**Affiliations:** a Electrochemical Innovation Lab, Department of Chemical Engineering, University College London London UK yuhan-liu@ucl.ac.uk rhodri.jervis@ucl.ac.uk; b The Faraday Institution Quad One Harwell Science and Innovation Campus Didcot UK; c Department of Chemistry, University College London 20 Gordon St. London WC1H 0AJ UK; d Istituto Officina dei Materiali (IOM)-CNR S.S.14 Km 163.5 Trieste I-34149 Italy; e Department of Biochemistry, University of Oxford South Parks Road Oxford OX1 3QU UK; f Department of Chemistry, University of Oxford, Inorganic Chemistry Laboratory South Parks Road OX1 3QR Oxford UK; g Advanced Propulsion Lab, University College London Marshgate 7 Sidings Street London E20 2AE UK

## Abstract

The reliability of surface characterisation in battery research is often compromised by the diagnostic techniques, which can induce unintended chemical changes during measurement. This is a particular concern for polyvinylidene fluoride (PVDF), which is a standard polymer binder used to maintain the structural integrity of battery electrodes. Such degradation creates measurement artefacts that can be easily mistaken for genuine electrochemical behaviour. This study utilizes X-ray photoelectron spectroscopy and kinetic modelling to investigate the X-ray-induced radiolysis of the PVDF binder. In addition, by comparing the behaviour of pure LiMn_*x*_Fe_1−*x*_PO_4_ powder to electrodes cast with PVDF, we demonstrate that the presence of the active material fundamentally alters how the binder decomposes, which triggers a consistent reduction of Fe oxidation states across all electrodes post-exposure. Specifically, the degradation is PVDF-concentration-dependent. The 2 wt% PVDF electrode displays a more rapid overall formation of LiF on the surface than the 10 wt% PVDF electrode, which shows an accelerated initial fluorine loss but a stabilised LiF content after 5 hours. These findings provide essential guidelines for interpreting spectroscopic data of battery electrodes, enabling researchers to properly distinguish measurement-induced artefacts from genuine electrochemical phenomena.

## Abstract 2

X-ray beam in a standard XPS measurement can rapidly trigger the PVDF degradation, which will trigger the subsequent formation of LiF on pristine, uncycled cathode surfaces.

## Graphical abstract

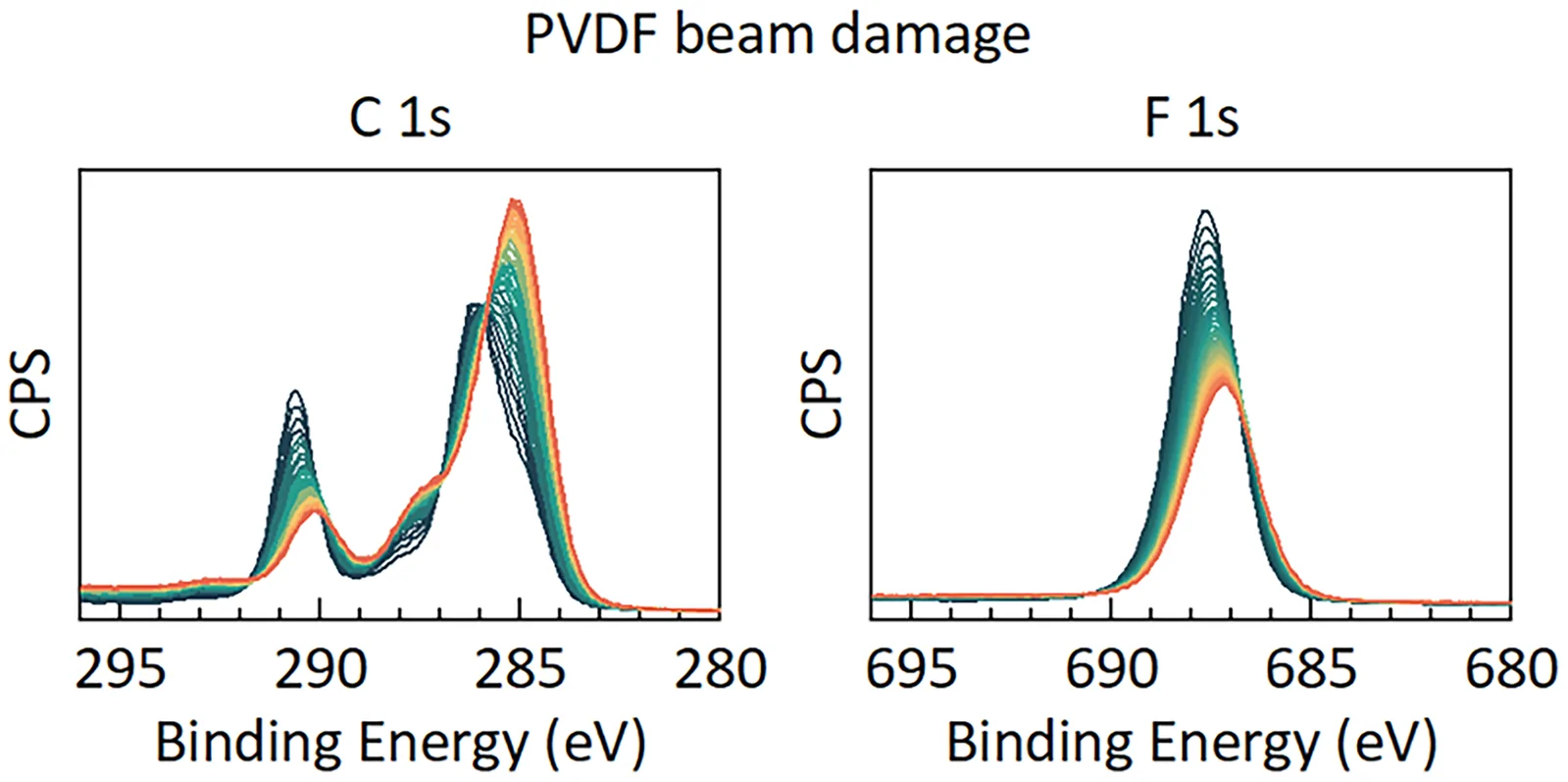

**Broader context**

The transition toward sustainable energy storage depends on our ability to design lithium-ion batteries that offer both extreme longevity and safety. Central to this mission is the precise characterization of the electrode interphase, where the presence of lithium fluoride is almost universally viewed as the definitive indicator of electrolyte salt decomposition. This study introduces a critical correction to this characterization paradigm by proving that the industry-standard PVDF binder serves as a significant and overlooked source of LiF through X-ray induced radiolysis. By demonstrating that the analytical technique itself creates the degradation products it is designed to detect, we provide a vital framework for ensuring data integrity across the energy research community. This finding is particularly consequential because it reveals how the catalytic nature of transition metal surfaces can accelerate these measurement-induced artifacts. While our current investigation centres on phosphate-based cathodes, the fundamental susceptibility of the PVDF polymer to radiation damage suggests that these findings are equally relevant to the study of the other electrodes. Researchers must now consider that the LiF detected during X-ray characterization may similarly stem from measurement-induced artifacts rather than purely from electrochemical cycles. Our work establishes a rigorous kinetic model that allows scientists to distinguish between these spurious signals and genuine interphase evolution, ensuring that the development of next-generation battery chemistries is guided by an accurate understanding of surface phenomena.

## Introduction

Lithium manganese iron phosphate (LiMn_*x*_Fe_1−*x*_PO_4_, LMFP) based cathode materials are central to the advancement of energy-dense lithium-ion battery cathodes made from earth abundant metals. In the fabrication of these cathodes, and many others, polyvinylidene fluoride (PVDF, –(CH_2_–CF_2_)_*n*_–) is the most widely used binder, selected for its excellent electrochemical stability, high chemical resistance, and strong adhesive properties.^1,2^ However, the long-term performance of these batteries depends on the formation of stable solid electrolyte interphase (SEI) and cathode electrolyte interphase (CEI), where lithium fluoride (LiF) is a key component of providing ionic conductivity and protective stability. Consequently, the precise measurement of LiF using surface-sensitive techniques, like X-ray photoelectron spectroscopy (XPS), is essential for understanding how these interphases evolve.

Within the battery research community, the appearance of a LiF peak at 685 eV in XPS is almost exclusively attributed to the decomposition of fluorine-containing electrolytes or additives, as the SEI or CEI component.^3,4^ However, this interpretation overlooks the binder as a potential fluorine source.^5^ This is particularly problematic when evaluating composite battery electrodes bound with PVDF. Previous studies have suggested that PVDF may not be stable under all conditions encountered during battery operation and characterisation.^6–9^ For example, LiF can be formed under irradiation on the electrode surface without cycling, due to LiPF_6_ decomposition as well as the reaction between PVDF and active materials.^3,5,8^ This suggests that a significant portion of F^−^ environment observed in XPS analyses may originate from a beam-induced artefact instead of electrolyte decomposition, presenting a significant challenge for accurate data interpretation, particularly the formation of LiF.

The potential for creating such analytical artefacts is particularly relevant during X-ray-based analysis, where sample damage can occur from the interaction with primary X-rays and the subsequent secondary electrons from photoionisation.^10^ Polymers, in particular the halogenated compounds, are known to be susceptible to such radiation damage which may not be limited to the surface.^6,11–16^ Polymers tend to degrade through bond scission and, subsequently, the loss of volatile compounds, *e.g.* HF and HCl. The chemical changes of the specimen from radiation damage can also lead to sample charging because of the loss of electronic conductivity, which further affects the XPS measurement.^17^ Hence, recent literature has begun warning about these radiolysis artefacts in XPS analysis.^3,6,18–21^ In 2024, Fantin *et al.* confirmed that high-flux synchrotron radiation triggers rapid PVDF radiolysis and subsequent LiF artifact formation on model LiNiO_2_ composite surfaces.^22^ However, that work and related studies concluded that under standard laboratory XPS parameters, this degradation remains practically negligible, since initial and final spectra are essentially identical.

This study directly confronts this analytical challenge and demonstrates that laboratory radiolysis can be significant for different PVDF–Li complex combinations. We prove that the X-ray beam in a standard XPS measurement can rapidly trigger the PVDF degradation and the subsequent formation of LiF on pristine, uncycled phospho-olivine cathode surfaces. The high vacuum environment isolates the radiolysis from the confounding variables from electrochemical cycling. We use this XPS-induced degradation as a novel tool to probe the formation and impact of LiF on interfaces. Surface characterisation was conducted using standard XPS parameters; however, to capture the full evolution of the chemical environment under X-ray irradiation, iterative high-resolution scans were performed over an extended duration. While such prolonged exposure time exceeds typical analytical timeframes, this approach was designed to isolate and quantify the kinetics of radiolysis. While previous studies have mapped these kinetics in pure materials, we demonstrate that the presence of the active material can fundamentally modulate the chemical path and kinetics of binder degradation. Because the most significant beam-induced radiolysis occurs during the initial stages of X-ray exposure, the artefacts observed here are not merely consequences of long-term radiation. This work, therefore, provides a foundational investigation into a critical analytical artefact, and the fundamental mechanisms of interfacial fluoride formation in lithium-ion batteries.

## Results and discussion

To study the radiolysis of active material, LMFP powder and an electrode (containing the same powder and PVDF) were subjected to iterative XPS measurement, during which high-resolution spectra were collected on the sample surface while the X-rays and flood gun simultaneously induce chemical changes. This approach allows us to monitor time-dependent changes under continuous X-ray exposure.

Fig. 1 compares the first and 25th cycle of C, F, Fe, and Mn core state spectra for both samples. Minimal differences were observed for neat LMFP powder after 15.42 hours of continuous X-ray exposure, as illustrated in the top row figures. The Fe and Mn 2p spectra exhibit characteristic satellite peaks at approximately 715 eV and 647 eV, respectively, which are indicative of their 2+ oxidation state. The overall spectral shape of LMFP powder appeared unchanged throughout the experiment (see Fig. S1 for the other elements), confirming its relative stability under prolonged radiation.^23^ The only perceptible variation was a minor decrease in the C 1s spectrum at 289 eV, which is attributed to the loss of surface oxygen under high vacuum environment of the XPS chamber, rather than X-ray-induced degradation of the LMFP powder (experiments on PVDF power under the vacuum without irradiation showed a similar minor difference, discussed in later section).

**Fig. 1:**
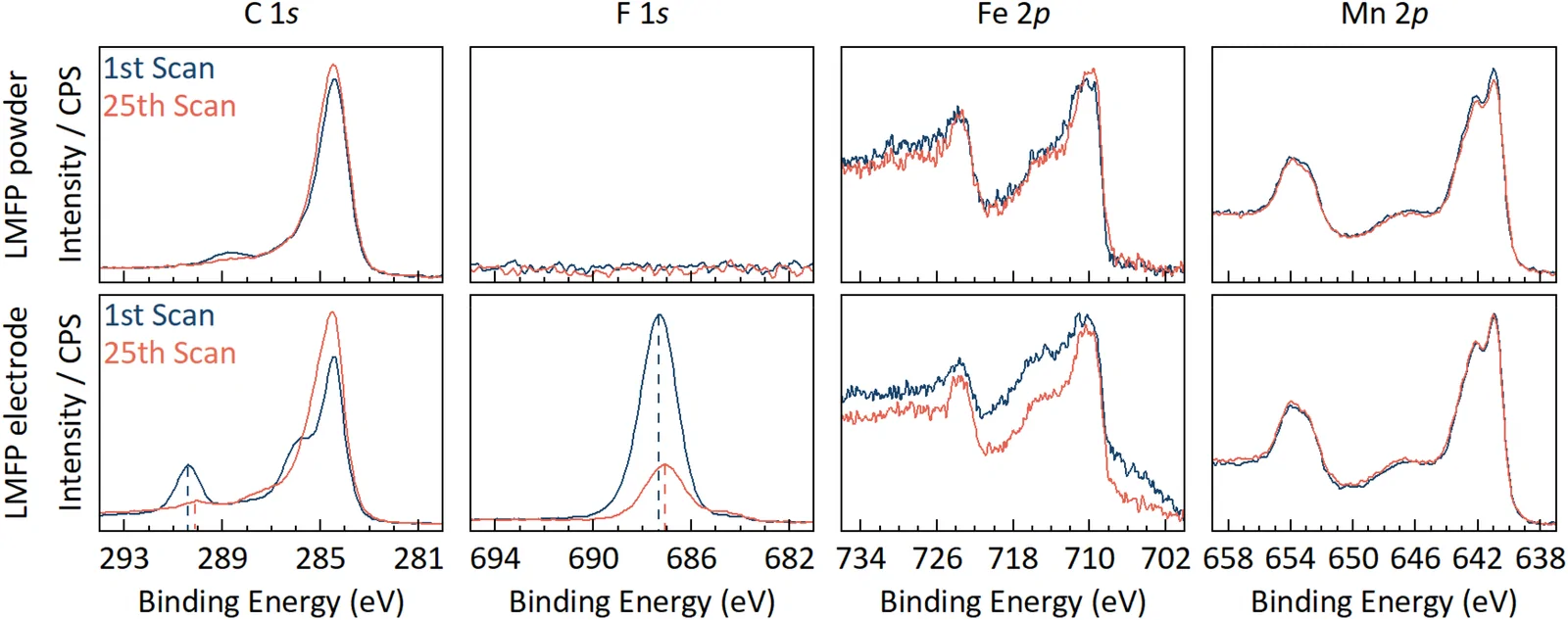
C 1s, F 1s, Fe 2p and Mn 2p core state spectra of the initial and the 25th scan of iterative XPS measurements of LMFP powder (top) and electrode (bottom). Baseline offset was applied to each spectrum to have the same intensities at lower BE. Binding energy was calibrated to the LMFP powder O 1s peak at 531.3 eV.

In contrast, the LMFP electrode which contains PVDF as binder, exhibited significant chemical changes throughout the measurement. A substantial decrease in the F 1s peak intensity was observed, alongside the corresponding loss of the peaks at 290–291 eV and 286.5–285.5 eV in the C 1s spectrum, pointing towards significant fluorine loss from the sample surface.^24^ Furthermore, the remaining peaks, as well as F 1s, exhibited a slight shift (0.4–0.5 eV) toward lower binding energy (BE). These combined observations provide clear evidence of PVDF backbone degradation under the X-ray beam.

Given that Mn represents 80% of the total transition metal composition, we would expect the spectral response to scale with the concentration of its constituents. However, while Mn and other elements remained unaffected (Li, O and P as seen in Fig. S1), the Fe 2p spectrum exhibited a notable decrease in its satellite peak intensity, as well as a subtle shift toward lower BE. In Fe XPS, the satellites arise from specific electronic transitions that are highly sensitive to the metal's valence state; a decrease here suggests a transition toward a slightly lower oxidation state after X-ray exposure.^25^ Notably, the Fe satellite intensity of the LMFP electrode prior to X-ray irradiation was subtly different from that of the pristine powder, likely due to surface oxidation during the electrode fabrication process. As X-ray exposure progressed, the Fe peak shape for the LMFP electrode evolved until it closely resembled the pristine state of the powder. This suggests that the radiolysis of the PVDF binder facilitates a reduction of surface-oxidized iron species to a state more closely resembling the pristine powder. These combined observations strongly suggest that the changes observed in XPS spectra are not intrinsic to the LMFP active material, but are a direct consequence of the PVDF binder degradation under X-ray exposure.

### PVDF degradation under X-rays

Since the radiolysis of the LMFP electrode only occurred in the presence of PVDF, PVDF powder was then analysed on its own using the same experimental conditions. An obvious PVDF degradation can be observed by optical microscopy after X-ray exposure, as shown in Fig. S2. Fig. 2(a and b) shows the evolution of the C 1s and F 1s spectra over XPS iteration scans on the PVDF powder surface, while Fig. 2(e) depicts peak fit analysis of the 1st and 41st scans of C 1s spectrum. The C 1s spectrum initially shows prominent peaks, which are assigned to C*–CF_2_ (286.3 eV) and C*–F_2_ (290.6 eV).^24^ Both peaks gradually decrease upon irradiation (590 s for one iteration scan), clearly indicating radiation-induced damage under XPS measurement. To reflect the evolution of PVDF, spectra were analysed without absolute BE calibration. The BEs of the C*–F_2_ and F 1s peaks give a linear correlation (Fig. S3), with a gradient (0.94) close to 1. This suggests that these chemical environments coexist within the same phase and undergo uniform charging shifts.^26^

**Fig. 2:**
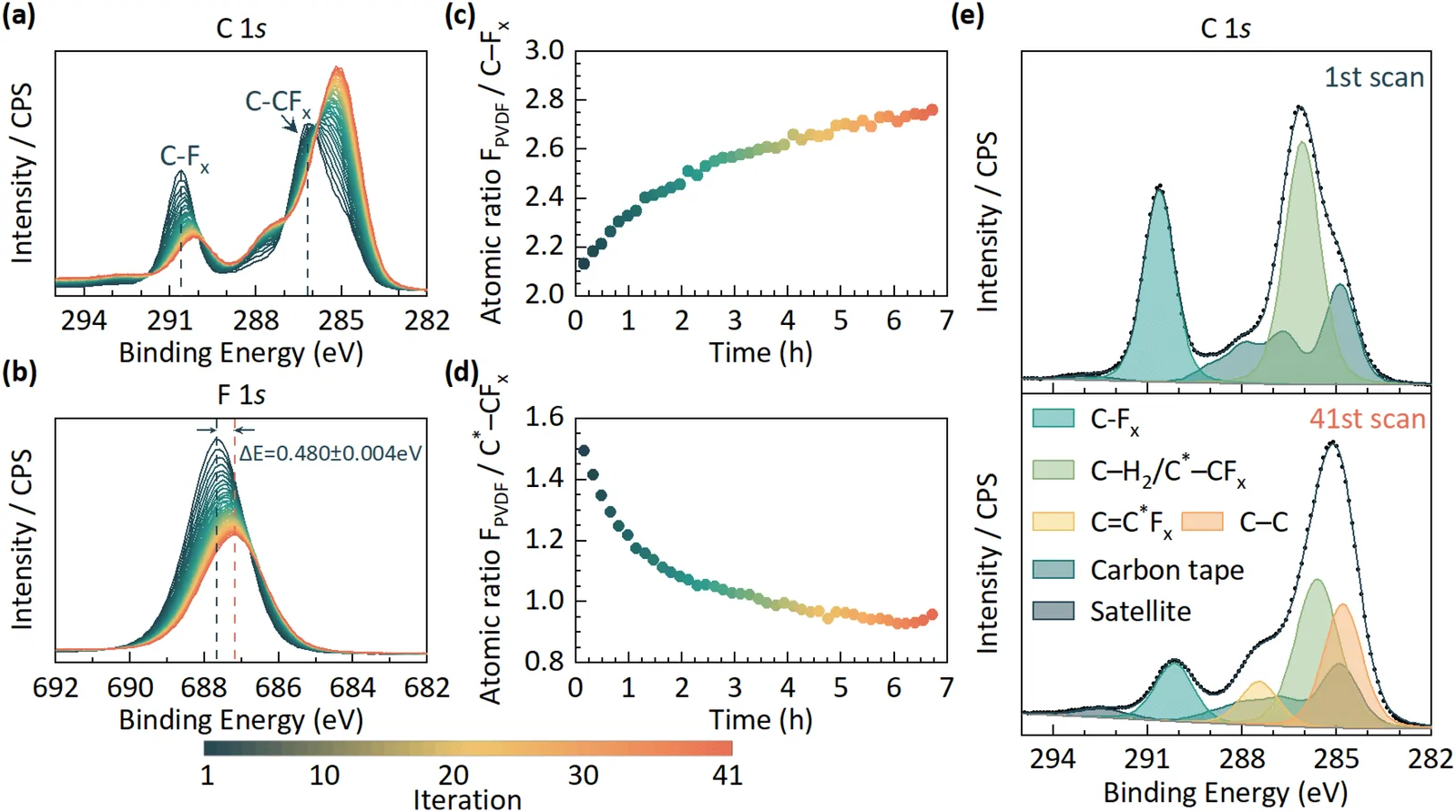
(a and b) XPS C 1s and F 1s core state spectral evolution of PVDF powder under continuous, iterative scans. (c and d) Temporal evolution of the 
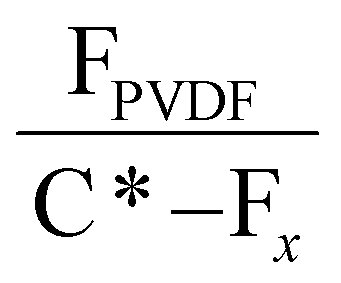
 and 
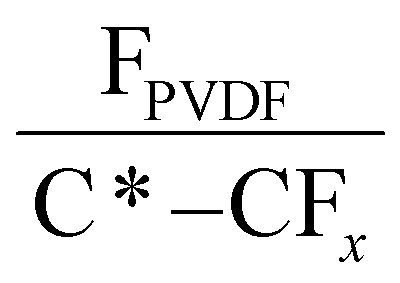
 atomic ratios as determines from peak fit analysis of the respective core state spectra. (e) Deconvolution of the C 1s spectra for the 1^st^ (top) and 41^st^ (bottom) scans. All spectra are presented without binding energy calibration.

Based on the observed changes in the XPS data, the radiolysis process is postulated to follow the mechanism of PVDF thermal degradation.^27^ After the primary scission of carbon–hydrogen/fluorine bonds from –(CH_2_–CF_2_)_*n*_–, the loss of HF results in forming polyenic sequences of –CH=CF– in the polymer chain. This leads to the distinct peak at 287.8 eV (C=C*F_*x*_), which is similar to fluorobenzene and also slightly moves to lower BE after iteration scans.^9,28,29^ Hence the peak at 293.0 eV is attributed to the π–π* carbon satellite, supported by the fact that its intensity increases concurrently, which contrasts with the behaviour of the PVDF peaks. To confirm the formation of double bonds, we performed Raman spectroscopy on PVDF powder after irradiation. Fig. S4 clearly shows the D and G band obtained on damaged PVDF powder.^30^

If PVDF was structurally perfect, the 
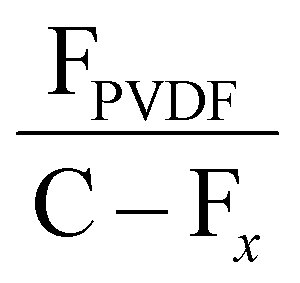
 and 
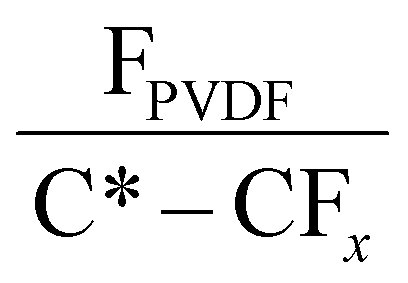
 atomic ratios should both be equal to 2. However, the initial values of 2.13 and 1.50, respectively, indicate that some initial degradation has already occurred. To account for this during peak fitting, the background carbon and oxygen environments from both the PVDF and the carbon tape were treated as a single combined baseline contribution. In Fig. 2(e), the peak labelled as ‘carbon tape’ encompasses both this background signal and the initial adventitious C–C surface contamination. We assumed that at this pristine stage, no intrinsic C–C signal from PVDF is detectable in the lower binding energy region, because the polymer backbone is fully fluorinated, localizing its carbon atoms strictly within the higher binding energy C*–CF_2_ and C*–F_2_ positions. As the PVDF backbone undergoes X-ray-induced radiolysis, the sequential loss of fluorine and hydrogen forces the polymer chain to crosslink and desaturate. This drives the formation of a new-generated carbon network, which is labelled as C–C in Fig. 2(e), with a corresponding energy offset to account for the observed energy shift in C*–F_2_ over iterations.

However, a fraction of these groups may subsequently undergo crosslinking reactions and further convert into C–F. Consequently, the observed C*–F_2_ environment transforms into a mixture of C–F and C–F_2_. Since differentiating C–F and C–F_2_ is challenging for the entire dataset – and this requires advanced deconvolution techniques or theoretical calculations beyond the scope of this work – they will be discussed as a single environment, and fluorine is labelled generally as F_*x*_ in the following discussion.^31,32^ The C–F_*x*_ represents the initial C–F_2_ groups and the following transformed carbon–fluorine species formed during the radiolytic degradation, while the F 1s peak (F_PVDF_) represents the total fluorine content within the analysed volume. As radiolysis progressed (Fig. 2(c and d)), C*–CF_*x*_ and C*–F_*x*_ peaks shift to lower BE in the C 1s spectrum, accompanied by the continuous change of both 
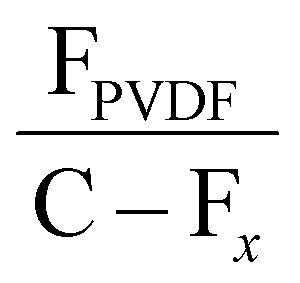
 and 
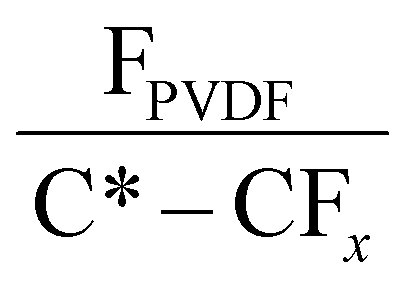
 atomic ratios, suggesting a constant PVDF structure degradation.

The evolution of the C*–F_*x*_, and F_PVDF_ (which represents the total fluorine environment) peak areas are shown in Fig. S5(a and b). To focus purely on the rate of change, the peak areas are normalized to the initial value, which allows for a direct comparison of the degradation across different samples or different spectra, even if their initial absolute intensities vary. The absolute intensity of an XPS peak can be influenced by sample loading, surface roughness, or slight variations in X-ray flux, which can cause variations in the absolute signal intensity that are not directly related to the degradation process, even within the same sample over extended measurement durations.^33,34^

Photodegradation is frequently modelled using first-order kinetics as *y*(*t*) = *A·e*^−*kt*^, where *A* is the amplitude, *k* is the rate constant, and *t* represents time.^6,35^ However, this model did not accurately describe our experimental data. As shown in Fig. 2(c and d), the degradation process appears to be two-step. If the PVDF degradation was a simple F-loss without new bond formation, the sum of the C*–CF_*x*_ and C*–F_*x*_ should be comparable to F 1s through the entire time of experiment. However, the loss of fluorine exceeded the carbon, as shown in Fig. S6, the sum of C*–CF_*x*_ and C*–F_*x*_ is higher than the F 1s intensity from the beginning, followed by a linear change during further iterations while F 1s drops more rapidly during the initial cycles. This suggests that the degradation mechanism involves a subsequent formation of other fluorine species on the surface. Hence, the C*–F_*x*_ observed should be separated as [C–F_2_](*t*) + [C–F](*t*), the concentration of C–F_2_ and C–F can be calculated as below, assuming both reactions are first-order and take place in a closed system:

C–F_2_:1
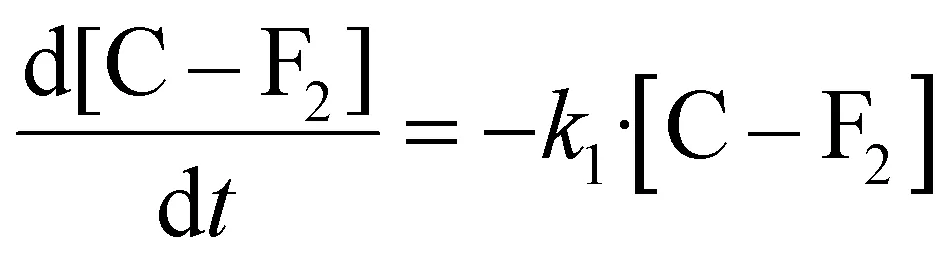

2 [C–F_2_](*t*) = [C–F_2_]_0_·*e*^−*k*_1_*t*^.

C–F:3
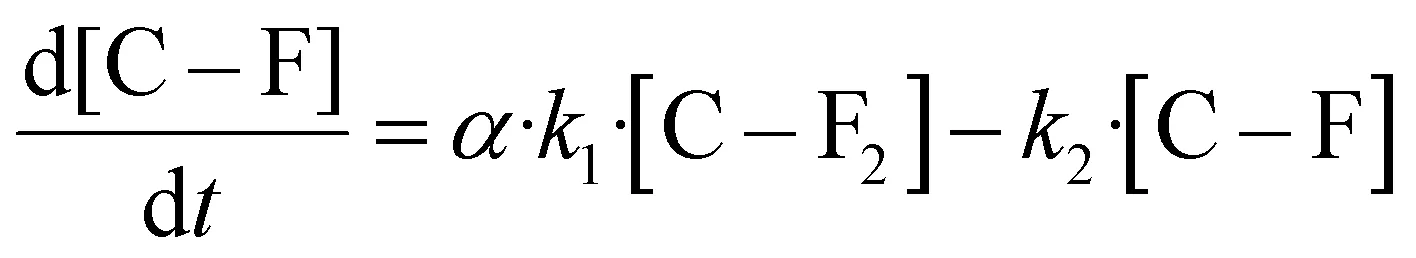
4
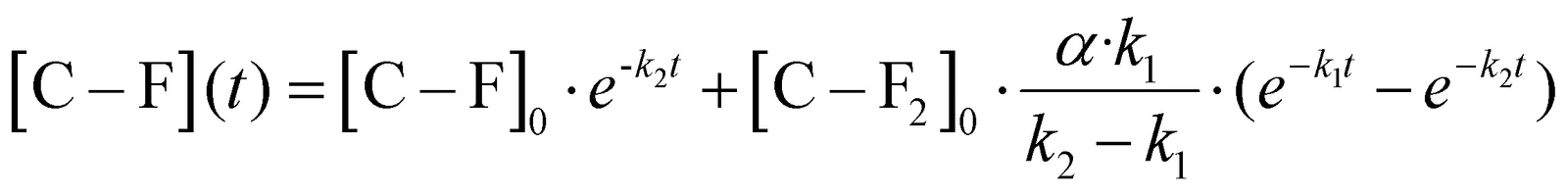
while the total fluorine should also consist of two parts: fluorine that is still chemically bonded to PVDF carbon, and fluorine that has been released but is still trapped within the sample matrix. Assuming only a fraction (*α*) of fluorine will be trapped, and it will leave the sample at a certain rate (*k*_loss_):

Bonded fluorine:

5 F_bonded_(*t*) = 2 × [C–F_2_](*t*) + [C–F](*t*).

Trapped fluorine:

6 F_released_(*t*) = (2 − α)·*k*_1_·[C–F_2_](*t*) + *k*_2_·[C–F](*t*)

7
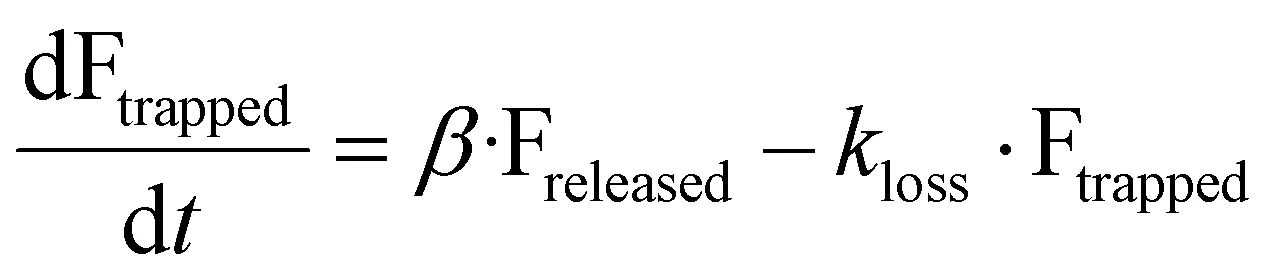

8 F_trapped_(*t*) = (F_trapped,0_ + *β*·F_released_(*t*))·e^−*k*_loss_*t*^.

Therefore, we can model the 
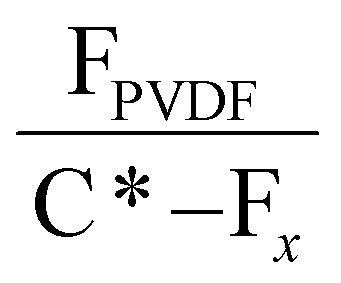
 ratio as:9
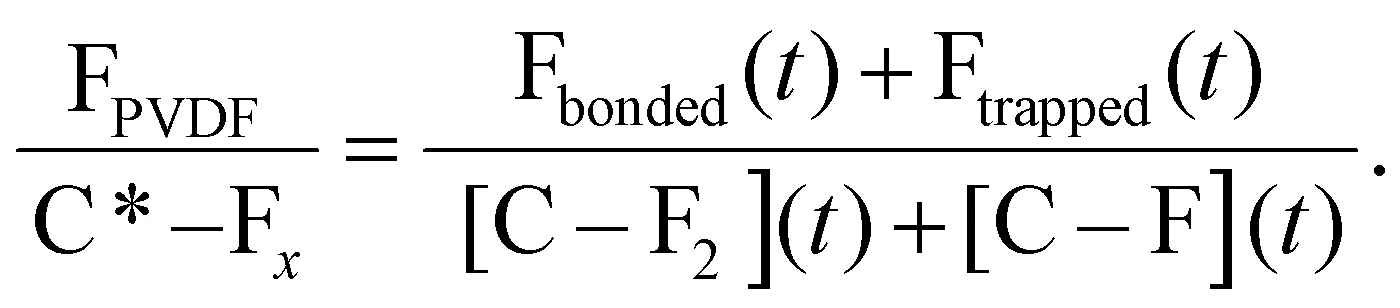


Rate constants can be calculated by applying this model to the observed data, where higher values represent a more rapid change. The initial, more rapid, exponential trend (governed by *k*_1_, 0.940 per h) suggests PVDF is susceptible to X-ray radiation and is likely to lose volatile compounds, which is accelerated by the ultra-high vacuum environment. The high *k*_1_ value indicates that the PVDF degradation is likely dominated by the faster scission of C–F bonds from C–F_2_ groups, leading to an obvious increase in C*=F_*x*_ (Fig. S7). These C–F_2_ groups partially bypass the more stable C–F structure, which has a slower radiolysis process (governed by *k*_2_, 0.058 per h). However, not all homolytic fluorine is immediately desorbed from the surface. The subsequent physical release of the fluorine atoms into the vacuum is a slower, potentially rate-limiting step.^6,29^ Essentially, this process creates a fluorine reservoir within the polymer matrix, thus 
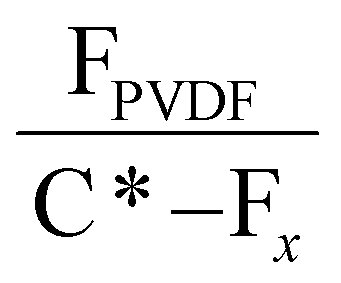
 increases significantly during XPS measurements.^9^ With the increasing desorption of fluorine, there is an increase at lower BE which should be assigned to carbon–carbon environments. Notably, when pristine PVDF powder is exposed to vacuum alone for 6.72 hours (the same time as the iteration measurements), the resulting changes to carbon–fluorine environments and F 1s peaks are negligible before or after undergoing iteration scans (see Fig. S8).

### LMFP electrode degradation under X-rays

The degradation of the LMFP electrode, consisting of LMFP, PVDF and a conductive carbon additive, was then compared with pure PVDF powder. Fig. 3(a–d) displays the C 1s and F 1s spectra for the LMFP electrode under iterative scans, and the peak fit of their 1^st^ and 25^th^ scans, respectively. Similarly, as seen in the PVDF powder, both C*–CF_*x*_ and C*–F_*x*_ exhibit a clear decrease and BE shift with increasing time (Fig. 3(a and c)), accompanied by a loss of F 1s peak intensity. This observation further confirms that radiolysis of PVDF results in fluorine depletion, regardless of whether it is in its isolated form or integrated within the composite electrode.

**Fig. 3:**
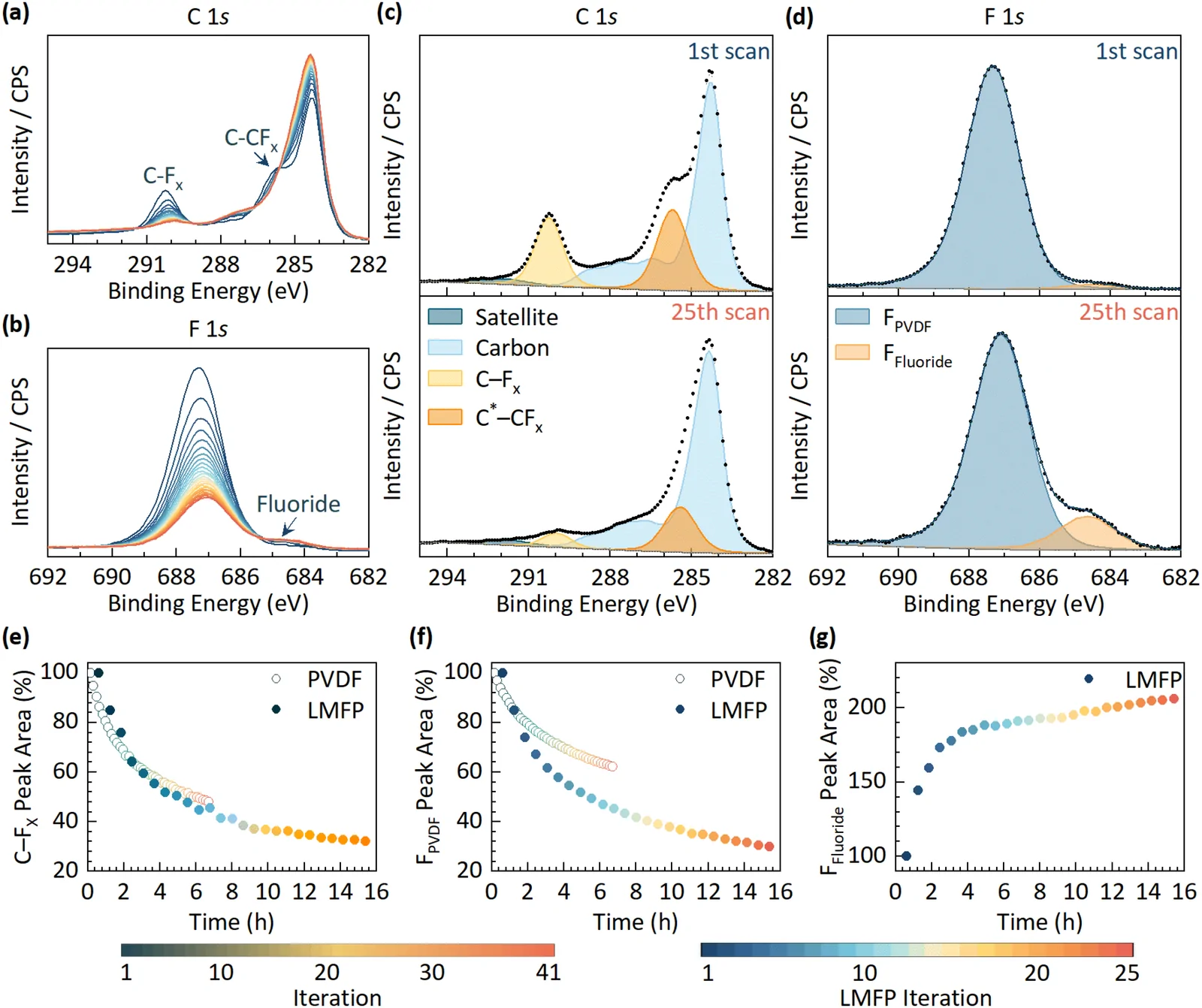
(a and b) XPS C 1s and F 1s core state spectral evolution of the LMFP electrode under continuous, iterative scans. (c and d) Peak fit of the C 1s and F 1s spectra for the 1^st^ and 25^th^ scans. (e and f) Relative peak area change of C–F_*x*_ and F_PVDF_ in PVDF powder and LMFP electrode. (g) Relative change of F_Fluoride_ peak area in the LMFP electrode. The binding energy was calibrated to O 1s peak at 531.3 eV.

To understand the chemical stability of the electrode under irradiation, the absorbed X-ray dose (energy absorbed per unit mass, Gy = J kg^−1^) was simulated using RADDOSE-3D, which computes spatially and temporally resolved dose fields in crystals undergoing X-ray radiation based on the beam and crystal characteristics.^36,37^ The average dose whole crystal (AD-WC) metric was determined to be the most appropriate according to the experimental setup.^38^ For an LMFP particle modelled as a 1 μm sphere, the simulation predicts the dose as 17.24 MGy per iteration (37 minutes), which increases ∼2% to 17.53 MGy when binder is introduced. This is because of the absorption of photoelectrons produced by the PVDF.^39,40^ It is important to note that these calculated numbers should not be interpreted as the real-world radiation dose, due to the highly simplified simulation that a lone active material crystal localized at the beam's centre where X-ray intensity is maximized. Instead, the utility of this model lies in its relative comparison regarding the addition of the polymer. The upward shift of relative X-ray absorption is physically constant because the surrounding polymer layer acts as a predictable capture zone for photoelectrons. However, this small relative dose increase is inadequate to explain the extent of radiolysis observed on the electrode surface.

For the pristine sample surface, the C*–F_*x*_ and F 1s peaks shifted to slightly lower BEs (290.2 eV and 687.3 eV) in the LMFP electrode compared to PVDF powder (290.6 eV and 687.6 eV). After 15.42 hours of irradiation, both peaks shift to a lower BE by 0.3 eV to 289.9 eV and 687.0 eV, respectively. Notably in the 1^st^ scan of F 1s, a small, distinct peak was also observed at a lower BE (*ca*. 684.5 eV), which is characteristic of inorganic fluoride species, such as LiF.^3,41^ This suggests either a minor amount of pre-existing fluoride on the electrode surface or an immediate, rapid fluoride compound formation upon initial X-ray exposure, leading to the lower BEs of the pristine sample. To separate these two fluorine environments, the fluoride peak is labelled as F_Fluoride_, while the PVDF binder environment in LMFP electrode is labelled as F_PVDF_. As depicted in Fig. 3(b and d), the amount of fluoride compound increases after iteration, evidenced by the raised F_Fluoride_ peak. The amount of fluoride can be modelled as:10
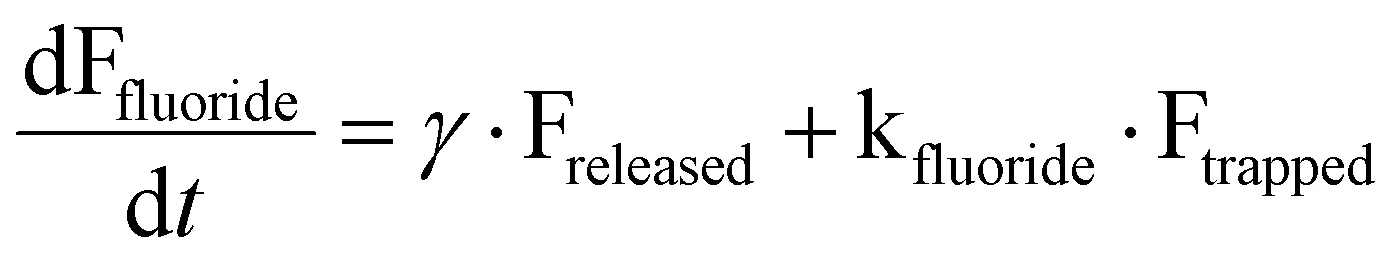
11
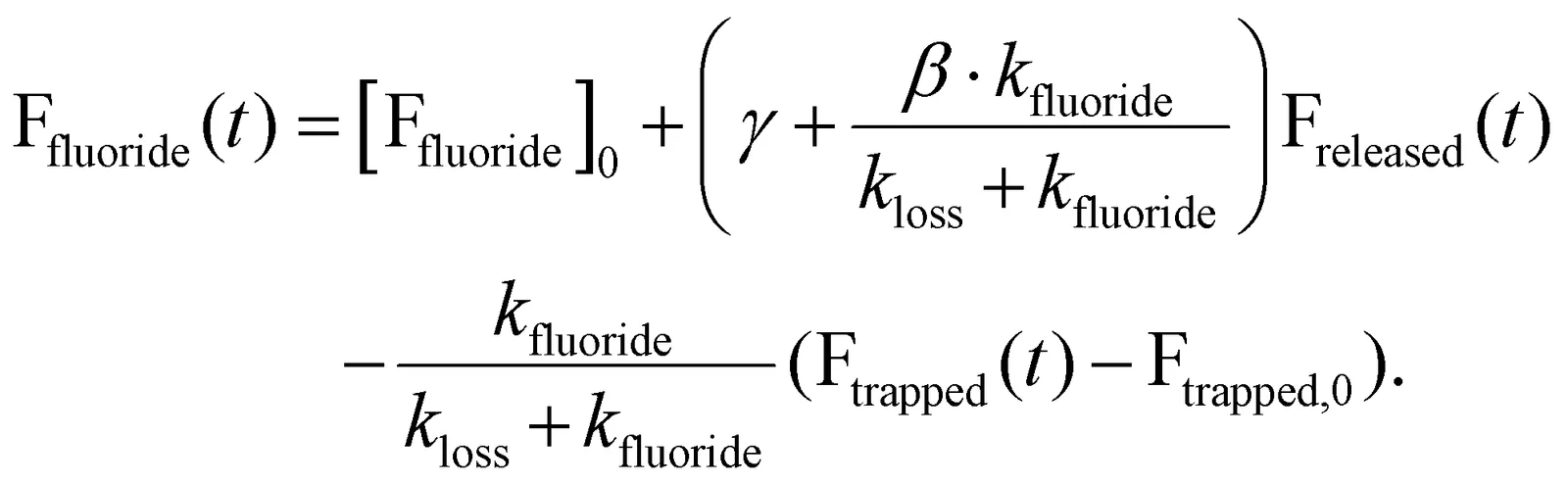


The F_PVDF_ and C–F_*x*_ peak area decrease of the LMFP electrode is shown in Fig. 3(e and f) with fitting parameters listed in Table S1. Although a slightly faster PVDF structural breakdown was observed in the LMFP electrode, attaining lower C*–F_*x*_ signal intensity than the pure PVDF powder after a similar amount of time, this is contradicted by the kinetic model. However this model only accounts the PVDF, and not the carbon and active material contribution, which is discussed later. The C–F_*x*_ bond in the PVDF structure degraded at a much lower rate (*k*_1_ = 0.588, *k*_2_ = 0.037) compared to its bulk powder, indicating a slower defluorination process compared to the neat PVDF material. Yet a more rapid decrease of F_PVDF_ was observed with a higher *k*_loss_ (0.071). Meanwhile, 
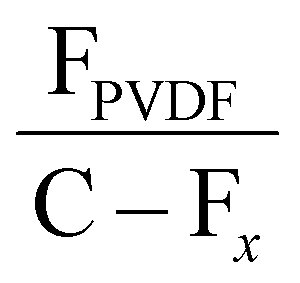
 exhibits a transient increase as neat PVDF powder before eventually declining (Fig. S9), whereas the 
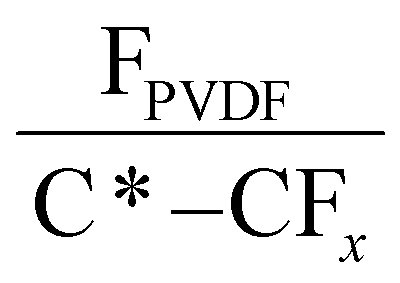
 atomic ratio decreases monotonically throughout the whole measurement.

The decrease in the F_PVDF_ peak intensity of the LMFP electrode is more obvious than that of C–F_*x*_, consistent with the rapid corresponding F_Fluoride_ peak growth (Fig. 3(g)). The inverse relationship between the loss of F_PVDF_ and the concurrent gain of F_Fluoride_ suggests that the defluorination of PVDF directly leads to the formation of inorganic fluoride compounds within the electrode. The continuous increase in F_Fluoride_ indicates that even under the vacuum environment, the liberated fluorine atoms react to form stable fluoride compounds on the electrode surface, which can then be confused with the SEI components. This highlights a significant X-ray-induced artefact that must be considered during XPS characterisation of PVDF-bound electrodes.

The rapid loss of F_PVDF_, contrasted with the slower defluorination of C–F_*x*_, suggests that the degradation of PVDF as an electrode binder is fundamentally different from the pristine polymer. Therefore, a complex interaction between the active material (LMFP), the carbon black additive, and the PVDF binder may occur during irradiation. It is known that chemical interactions at the conductive carbon–PVDF interface could weaken C–F bonds prior to irradiation.^42^ The presence of the carbon black additive provides high surface area, which may facilitate the efficient emission of secondary electrons under radiation. These electrons are absorbed by the surrounding PVDF, effectively increasing the local radiation dose at the polymer–carbon interface. This increased electron density promotes bond scission within the polymer matrix, leading to faster degradation than would occur in a carbon-free system.^43,44^

To experimentally validate this mechanism, we performed a control experiment using a binary mixture of PVDF and conductive carbon black (C65) in the absence of LMFP particles. The time-resolved radiolysis kinetics of this binary mixture revealed the highest rate parameters (*k*_1_ = 1.684, *k*_2_ = 0.073, see Fig. S10 and Table S1) across all evaluated systems. This rapid degradation directly confirms that the high-surface-area conductive carbon network acts as an exceptionally efficient emitter of secondary electrons, accelerating homolytic C–F bond scission. Under identical XPS operational parameters, the total incident photon flux density per unit surface area delivered to the sample should remain constant across all experiments. However, the effective energy dose received by the PVDF is dependent on the surrounding environment. When carbon black is added, the rapid secondary electron emission from the high-surface-area carbon network yields the higher degradation rate.

Interestingly, the fact that the full composite LMFP electrode exhibits slower kinetics than this PVDF + C65 mixture points to a competitive mechanism. In the full composite LMFP electrode, the direct photon dose received by the PVDF binder per unit volume is substantially lower. Because heavy transition metals like iron and manganese possess a fundamentally higher X-ray absorption cross-sections at 1486.6 eV. Consequently, these particles physically interrupt the continuous contact between the PVDF and the carbon black network while acting as a highly absorbing shield.

Beyond this physical dose attenuation, the chemical interplay at the active interface provides an additional thermodynamic driving force. The presence of Li^+^ ions allows immediate, irreversible reaction of liberated fluoride species originating from the PVDF scission to form LiF.^8,41^ Both carbon-driven secondary electron emission and this rapid interfacial LiF formation thermodynamically pull the polymer degradation forward, simultaneously inducing a coupled reduction of surface iron species to a lower oxidation state.^8,45^ Furthermore, localised heating effects or differences in X-ray energy absorption within the composite structure could also facilitate the removal of degradation products and preventing re-fluorination.

The correlation between the simulated dose enhancement and the observed spectra changes under X-ray exposure confirms that the growth of the F_Fluoride_ peak is a beam-induced artefact. The same X-ray dose model used for LMFP reveals a higher absorption dose for LFP (17.96 MGy for single LFP particle and 18.26 MGy with binder). This relative higher absorption of LFP crystal is a physical consequence of the higher mass absorption of iron for Al Kα radiation than manganese.^46^ Both models support our observation that PVDF degrades under irradiation while the existence of cathode material can directly modulate the residue decomposition compound. Ultimately, we provide the justification for the rapid LiF formation and peak shifting observed experimentally, reinforcing the need to consider X-ray-induced artefacts when interpreting the XPS data of composite electrodes.

### Does higher PVDF content result in more radiolysis?

To establish the practical field-wide utility of our work, we sought to investigate how the electrode composition influences the beam-induced artefact. In battery research, minimizing inactive components, such as binders and conductive carbon additives, is critical to achieve high energy densities. However, the analytical consequences on surface-sensitive characterization remain largely unexplored. Since our primary results indicated that the LFP framework naturally exhibits a higher absorption than an LMFP crystal, here we strategically switch to LFP cathodes to amplify the observable differences in radiolysis.

Two composite electrodes were prepared: one with 2 wt% PVDF (5 wt% of C65, 93 wt% LFP), and another with 10 wt% PVDF (5 wt% of C65, 85 wt% LFP). Dose simulation shows minor differences (<0.1%) with various binder concentrations; however, the experimental findings diverge from this result. While the initial C 1s spectra showed lower carbon–fluorine signal for the 2 wt% electrode as expected (Fig. 4(a and b)), the subsequent evolution of the fluorine environment revealed that the radiolysis-induced formation of LiF is not linearly dependent on binder content.

**Fig. 4:**
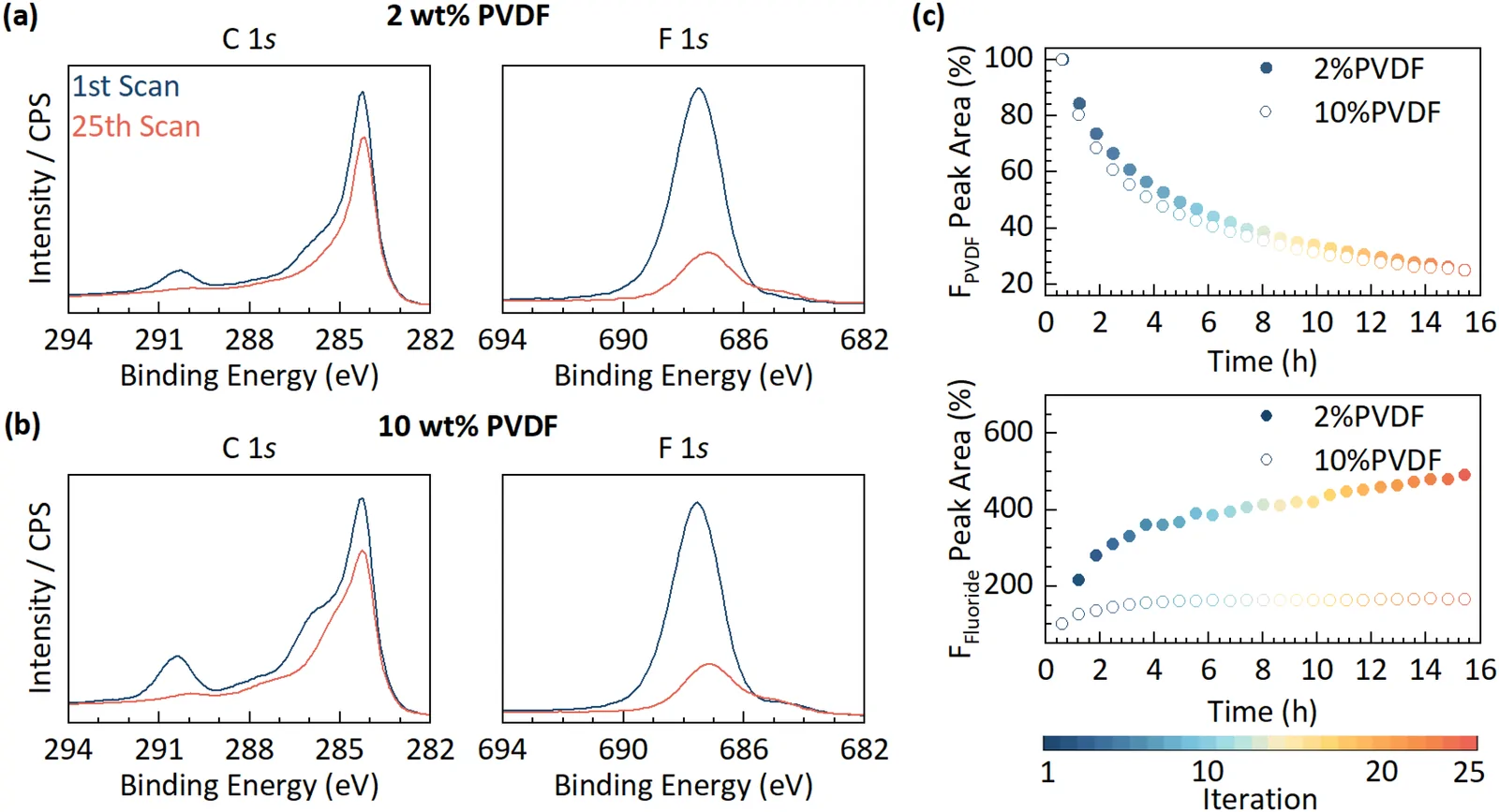
(a and b) The 1^st^ and 25^th^ scans of C 1s and F 1s spectra of LFP electrode using 2 wt% and 10 wt% PVDF. (c) Relative changes of F_PVDF_ and F_Fluoride_ peak area in LFP electrode using 2 wt% and 10 wt% PVDF. The binding energy was calibrated to the O 1s peak at 531.3 eV.

Although a comprehensive kinetic model was initially applied, the low spectral intensity of the C*–F_*x*_ peaks as well as the complex C 1s environment introduced significant ambiguity to distinguish the difference between two LFP electrodes. In contrast, the F 1s region offers a simpler chemical environment with a wide separation in the F_PVDF_ and F_Fluoride_ BEs. Therefore, only the change of their F_PVDF_ and F_Fluoride_ peaks were analysed and are depicted in Fig. 4(c). Despite a faster initial reduction of the 10 wt% PVDF sample, both electrodes ultimately converged to a similar relative signal intensity of F_PVDF_. A clear F_Fluoride_ peak was present in the pristine 10 wt% PVDF electrode, while its growth plateaued after approximately ∼5 hours of exposure. In contrast, the 2 wt% electrode showed a continuous increase in LiF formation (Fig. 4(c)).

We hypothesize that this accelerated growth in the low-binder electrode is driven by the higher interfacial area-to-volume ratio and surface passivation. In the 2 wt% sample, the PVDF likely exists as a thinner layer that a greater proportion of the binder is in direct contact with the LFP surface. Consequently, LiF can be more efficiently generated at the interface. In the 10 wt% sample, however, a smaller fraction of the binder is in contact with the active material. More importantly, the presence of LiF in the pristine 10 wt% electrode likely passivates the surface, suppressing further reaction and leading to the observed plateau in LiF content.

Li 1s, Fe 2p and 3p, O 1s and P 2p spectra are depicted in Fig. S11. Consistent with previous observations in the LMFP (Fig. 1), both LFP electrodes began in a higher oxidation state and reduced toward an identical Fe^2+^ state upon irradiation. Notably, the 10 wt% PVDF electrode exhibits a higher initial oxidation state compared to the 2 wt% sample. In addition, a minimal peak shift to higher BE is observed in P 2p (approx. 0.05 eV). While subtle, this shift is persistent across iterations in the LMFP electrode. We attribute this to the higher relative concentration of Fe in the LFP electrodes, which alters the local chemical environment of the phosphate groups.

## Conclusion

This work establishes a fundamental understanding of X-ray-induced artefacts in electrode materials. We clarify the nature of beam-induced damage at the interface, providing a necessary framework for distinguishing radiolysis changes from inherent material properties. A primary implication of this work is the observation of LiF on electrode surfaces that have neither been electrochemically cycled nor exposed to a fluorine-containing electrolyte. While the presence of F_Fluoride_ in F 1s spectrum is commonly identified as the decomposition of salts (such as LiPF_6_) or electrolyte additives, our results demonstrate that the PVDF binder itself serves as an alternative fluorine source. This, therefore, means that the use of XPS to understand the nature of SEI and CEI layers can be clouded by additional LiF signal from beam damage of the materials. Kinetic analysis supports a model where liberated fluorine from the PVDF matrix rapidly reacts to form surface LiF. Interestingly, while the fluoride signal remains relatively stable in electrodes with 10 wt% PVDF, the 2 wt% PVDF electrodes exhibit rapid LiF formation, suggesting that lower binder concentrations are more susceptible to localised radiation damage.

These observations highlight the risk of significant analytical artefacts in XPS measurements, which results in data misinterpretation if unaddressed. Specifically, we demonstrate that *in situ* LiF formation can occur even in the absence of fluorine-containing electrolytes, purely as a result of beam-induced degradation. It is also worth noting that factors such as the instrument model, X-ray flux, spot size, vacuum levels, and charge neutralisation settings can all directly impact the rate of radiolytic degradation; hence, the kinetic parameters are often unique to specific experimental setups and should be compared with caution. To mitigate these effects, we suggest the community adopt best practices such as implementing data acquisition on ‘fresh-spots’, utilising time-dependent scans to monitor for peak evolution, and minimising the spot size or number of scans where possible, when they use lab XPS systems. Ultimately, by characterising these artefacts, this work provides a framework for researchers to confidently distinguish between genuine electrochemical phenomena and instrumental effects, ensuring more robust and reproducible surface analysis in battery science.

## Methodology

Lithium manganese iron phosphate electrode was prepared using 92.5% LMFP (80% Mn, Aleees), 2.5% conductive additive carbon black (Alfa Aesar C65) and 5% PVDF (Solef). For the binary control sample, PVDF and C65 were mixed in a 1 : 2 weight ratio. Lithium iron phosphate (LFP) electrode slurries were prepared using LFP (Aleees), conductive additive carbon black (C-Nergy Super C45, Timcal) and polyvinylidene fluoride binder (PVDF, Solef). Two LFP formulations were prepared, with their ratios given in Table S2. PVDF was added to stirred NMP to prevent agglomeration and the mixture was stirred until dissolved to give an 8 wt% solution. For each formulation, carbon black was added to a portion of this binder solution, and mixed in a planetary mixer (Thinky ARE-250, Intertronics) for 1 minute at 500 rpm and 5 minutes at 2000 rpm. LFP was then added alongside the additional NMP required to give 45% solids content, and mixed for 10 minutes at 2000 rpm with a subsequent 3 minutes degassing at 2200 rpm in the planetary mixer. Electrode slurries were coated onto carbon coated aluminium foil, while the control sample was coated onto aluminium foil directly, with a doctor blade (3600/4, Elcometer) with a gap set to 150 µm and transferred to a hot plate to be dried at 60 °C for 5 hours. Dried foils were calendered with an electric cold roller press (MSK-HRP-MR100DC, MTI), with the roller gap set to the thickness of the current collector added to 50% of electrode thickness.

X-ray photoelectron spectroscopy (XPS) was measured using a Thermo Scientific NEXSA spectrometer, which uses a monochromatic Al K_α_ X-ray source (*hν* = 1486.6 eV), a hemispherical analyser and two-dimensional detector. Measurements were conducted with a 400 μm spot size, using a dual beam flood gun (electron and Ar^+^ ion) with 100 mA current. Pass energy was set to 200 eV for the survey spectra, and 40 eV for core levels. The same experimental conditions were applied to all LMFP electrodes and powders to ensure the total X-ray exposure, while the scan numbers are increased for LFP electrodes accordingly.

The CasaXPS software package was used for XPS spectra analysis, and all spectral fitting is performed with Tougaard background.^47^ To accurately capture the real-time evolution of the isolated polymer phase, pristine PVDF powder datasets were processed without binding energy calibration. For all composite electrodes, standard charge correction using the adventitious C 1s peak at 284.8 eV was bypassed, because we are concerned that the dynamic chemical breakdown of PVDF may cause continuous, beam-induced shifts. Since the lattice framework of the active material remains highly invariant and resilient against irradiation, the inorganic oxide O 1s peak at 531.3 eV (obtained from pristine LMFP powder sample), was used as an internal standard for calibration. To maintain the physical meaning and eliminate software optimization ambiguity, the full width at half maximum (FWHM) values for all individual sub-components were constrained to an identical value within a given C 1s dataset, except for the fundamentally broader high-energy π–π* satellite peak. The detailed raw peak areas and binding energy positions calculated from this constrained fitting procedure are given in Tables S4 and S5. The atomic ratios are calculated from the deconvoluted peak area divided by their relative sensitivity factors.

The absorbed X-ray dose was simulated using RADDOSE-3D v5.0.1047^36,37,40^ with input parameters summarized in Table S3. The small molecule feature is used to accommodates the input of the full chemical composition of the irradiated crystal. The feature of fluorescent escape is included because fluorescent photons are emitted from metal elements which reduces the absorbed dose. To investigate the effect of the PVDF binder, the surrounding material feature is used with the theoretical PVDF density 1.78 g cm^−3^ and conductive carbon density 1.86 g cm^−3^. The spot size of the XPS instrument was previously determined by Fernando *et al.* in 2021, and photon flux information was acquired directly from the supplier.^38^

## Conflicts of interest

The authors declare no conflict of interest

## Supplementary Material

EB-002-D6EB00103C-s001

## Data Availability

Supporting data are provided in the supplementary information (SI). Supplementary information is available. See DOI: https://doi.org/10.1039/d6eb00103c. The XPS data supporting the discussion is available from the following repository: https://doi.org/10.5522/04/33271128.
