# Supplementary material for: Radiolysis study on PVDF binder – how does it affect the X-ray photoelectron spectroscopy characterisation of phospho-olivine electrodes?
Source: EES Batter. 2026 Aug 7;2(5):1657–67. doi: 10.1039/d6eb00103c (PMC13655890; doi:10.1039/d6eb00103c)
Supplement: Supplementary file 1 [file EB-002-D6EB00103C-s001.pdf]

Supplementary Information (SI) for EES Batteries.
This journal is © The Royal Society of Chemistry 2026

Supporting information

to

Radiolysis study on PVDF binder – how does it affect the X–ray photoelectron spectroscopy characterisation of phospho-olivine electrodes

Yuhan Liu[1,2,3,7], Prajna Bhatt[2,4], Will Dawson[1,3,7], Alice Llewellyn[1,3], Joshua Dickerson[5], Robert Palgrave[2], Anna Regoutz[6], James Robinson[1,3,7], Rhodri Jervis[1,3,7]

[1] Electrochemical Innovation Lab, Department of Chemical Engineering, University College London, London, UK
[2] Department of Chemistry, University College London, 20 Gordon St, London, WC1H 0AJ, UK
[3] The Faraday Institution, Quad One, Harwell Science and Innovation Campus, Didcot, UK
[4] Istituto Officina dei Materiali (IOM)-CNR, S.S.14, Km 163.5, Trieste I-34149, Italy
[5] Department of Biochemistry, University of Oxford, Inorganic Chemistry Laboratory, South Parks Road, Oxford OX1 3QU, UK
[6] Department of Chemistry, University of Oxford, South Parks Road, OX1 3QR, Oxford, UK
[7] Advanced Propulsion Lab, Marshgate, 7 Sidings Street, University College London, London, E20 2AE, UK

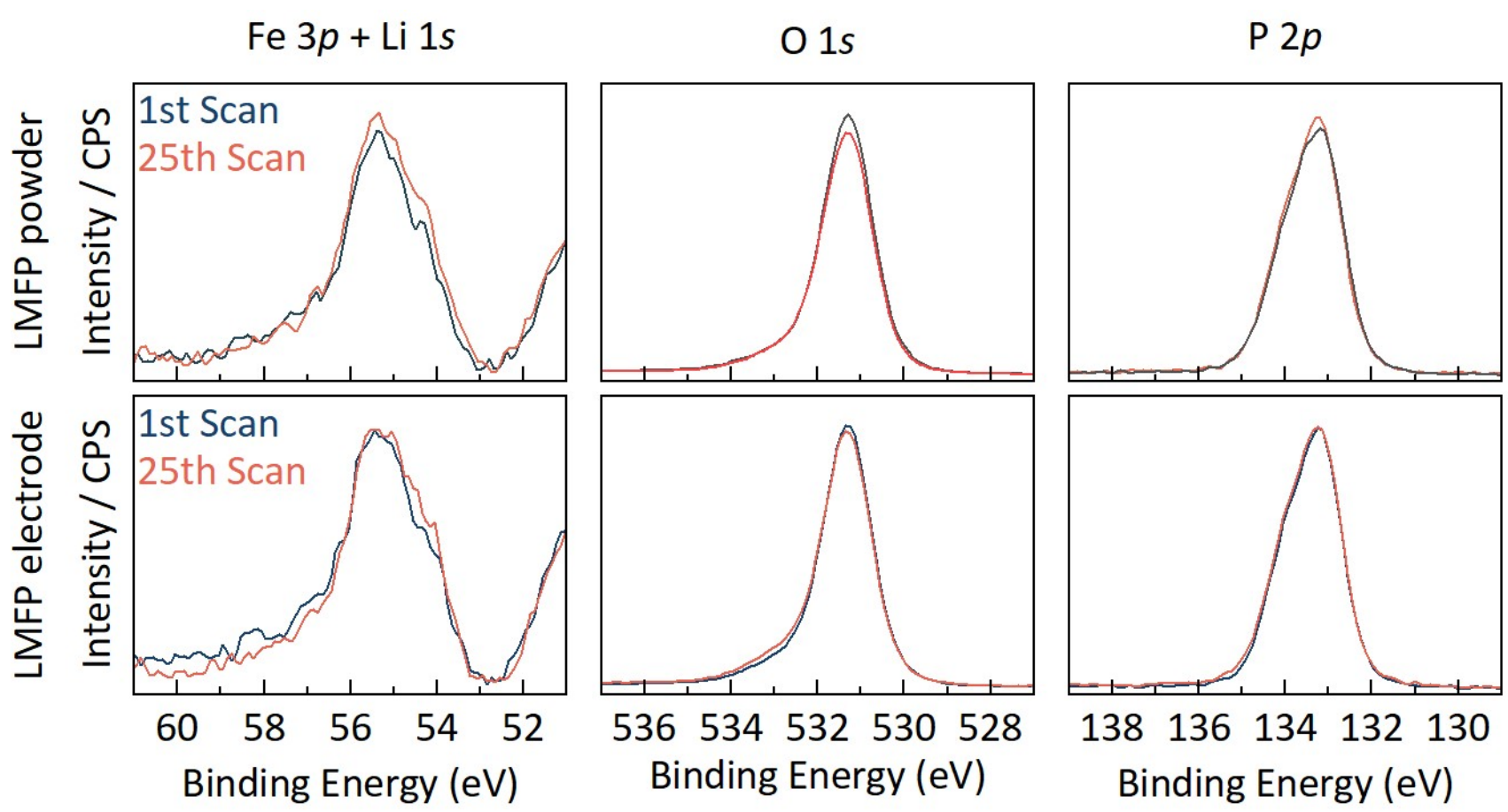

Figure S1 Li 1*s*, Fe 3*p*, O 1*s* and P 2*p* core state spectra of LMFP powder and electrode under continuous, iterative scans. The peak at *ca*. 51 eV is I 4*d* due to cross contamination during sample preparation. The overall spectral shape unchanged throughout the experiment

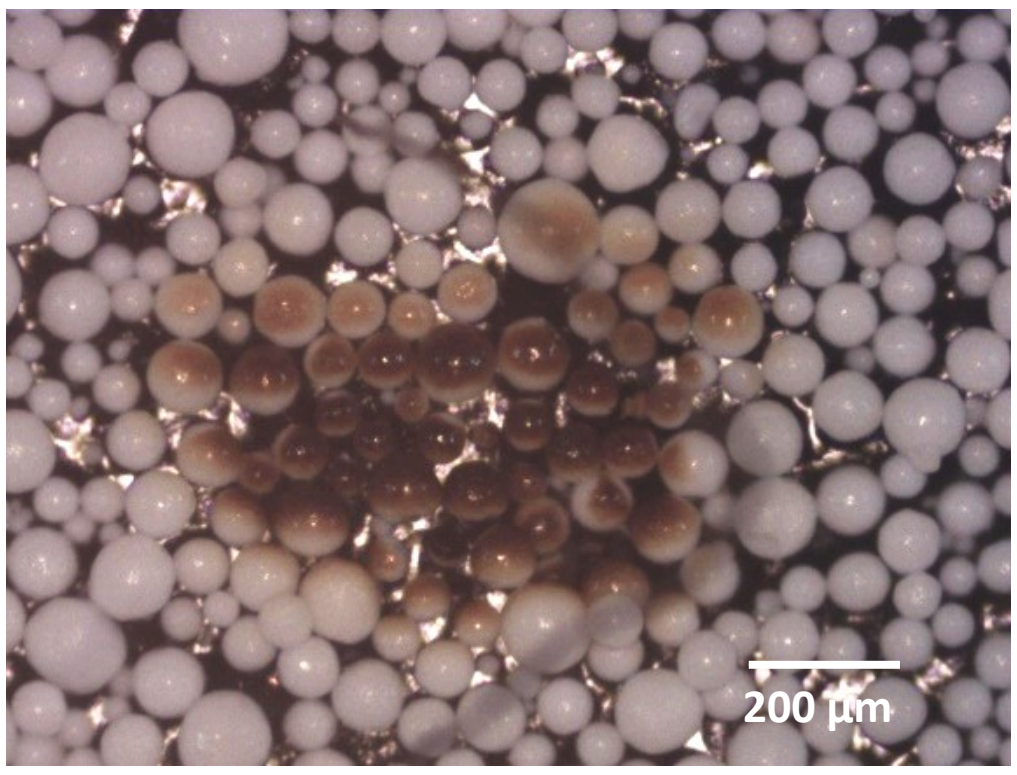

Figure S2 Beam damage effects on the PVDF powder showing discolouration. Image is taken by a 20× optical microscope.

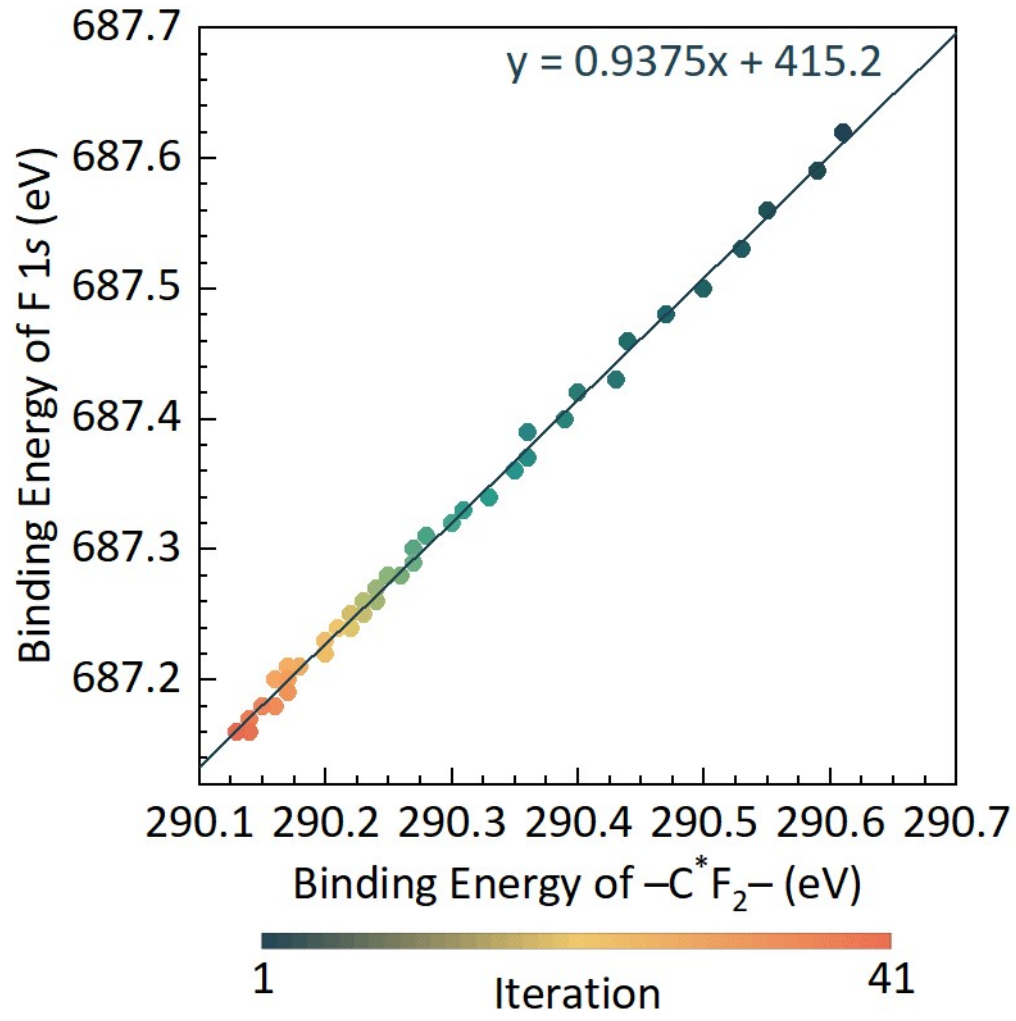

Figure S3 A linear binding energy correlation between $C^*–F_2$ and F 1*s* peaks with a gradient close to 1, indicating they are from the same phase.

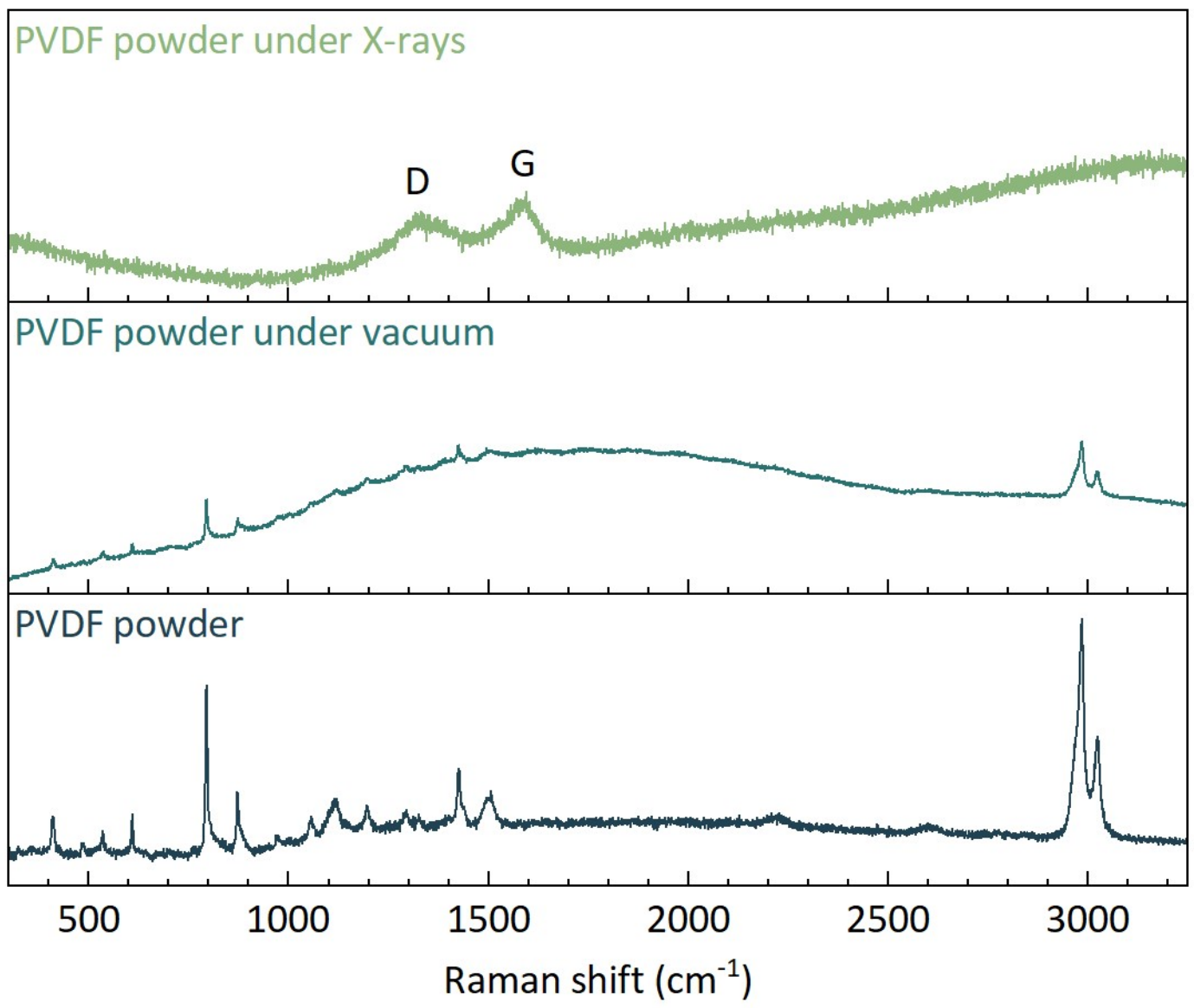

Figure S4 Raman spectra for PVDF powder, under vacuum and X–rays after 6.72 hours on carbon tape. The D and G band obtained after irradiation clearly indicates the existence of carbon double bonds.

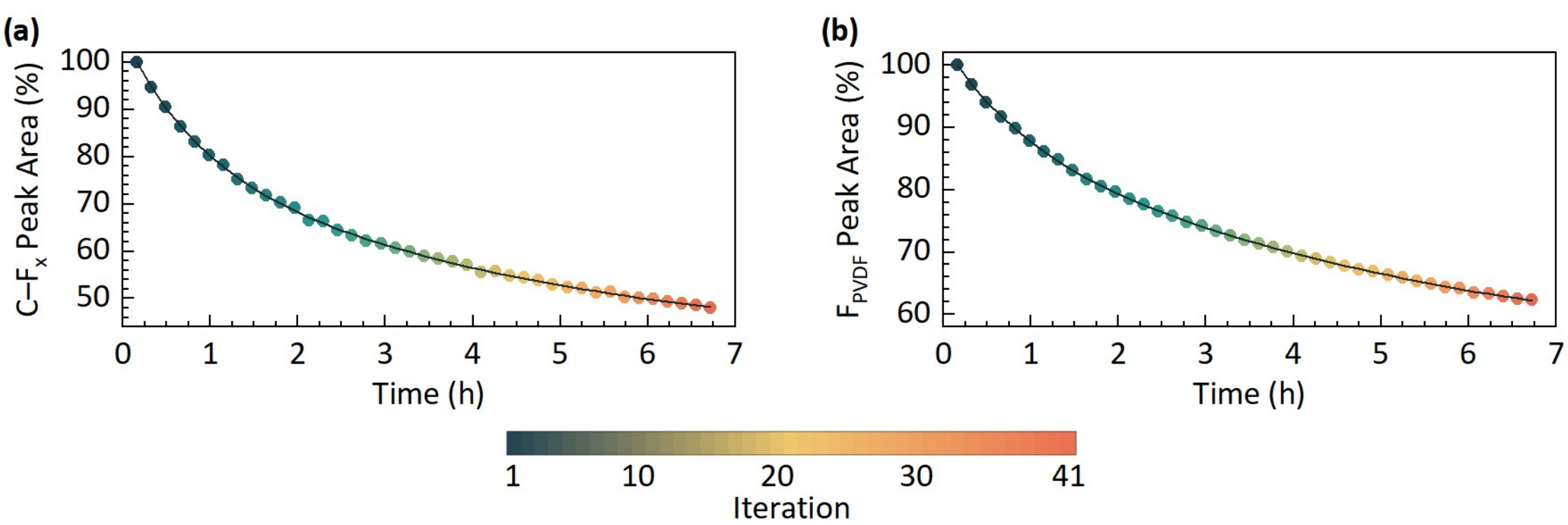

Figure S5 Temporal evolution of (a) $C–F_x$ and (b) F 1*s* peaks from XPS peak fit analysis relative to their first iteration scans.

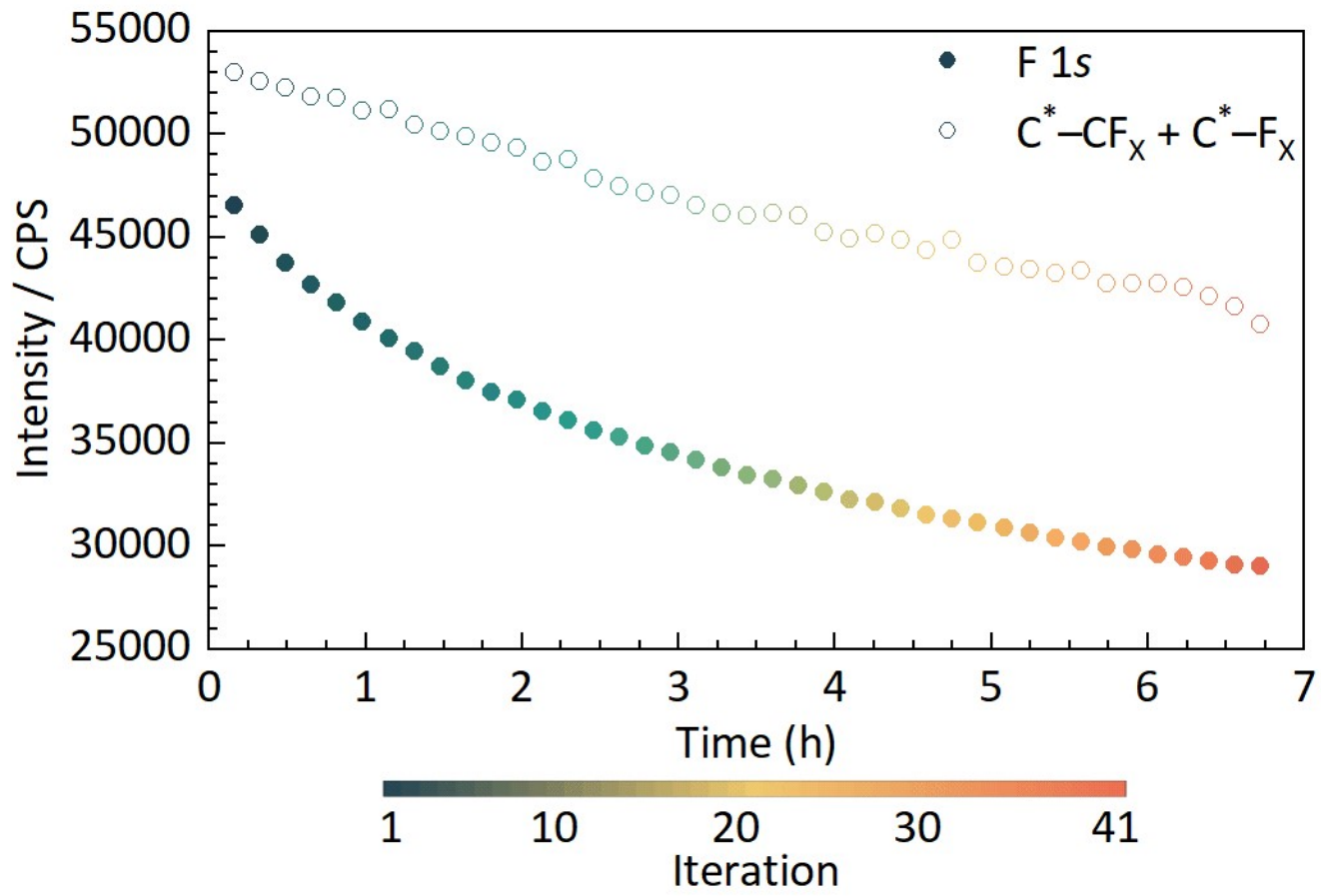

Figure S6 The F 1*s* compared to the sum of $C^{*}–F_x$ and $C^{*}–CF_x$. This suggests that the degradation mechanism involves a subsequent formation of other fluorine species on the surface. Peak areas were normalized by their respective relative sensitivity factors.

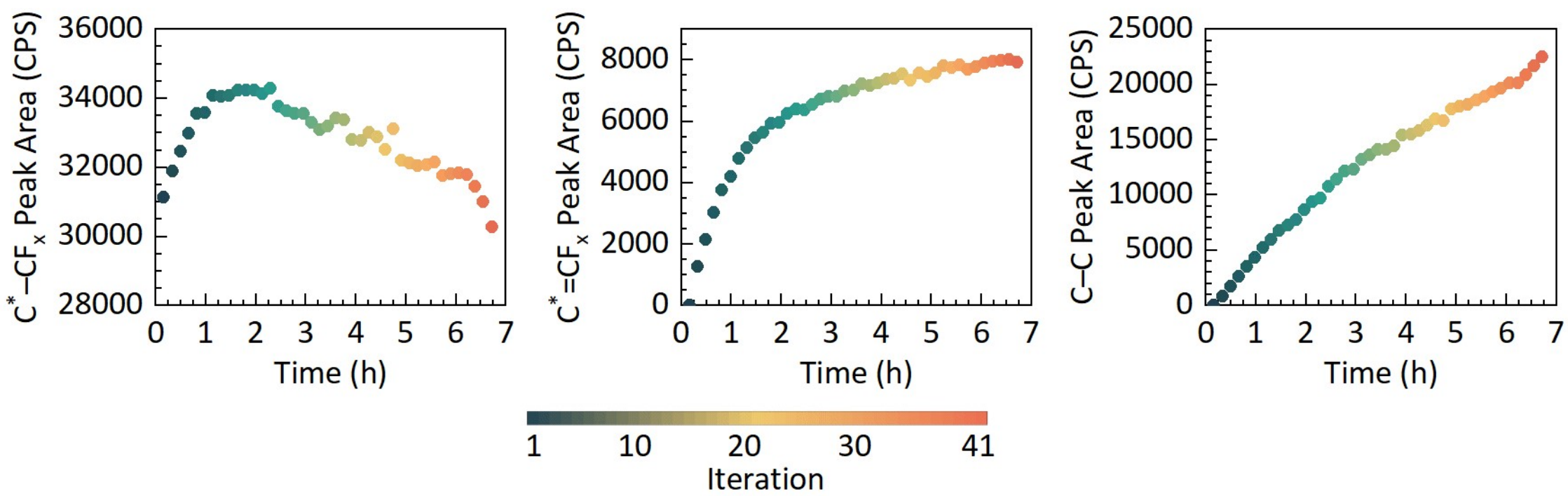

Figure S7 Temporal evolution of $C^{*}–CF_x$, $C^{*}=CF_x$ and C-C peak areas under continuous, iterative scans. Peak areas were normalized by their respective relative sensitivity factors.

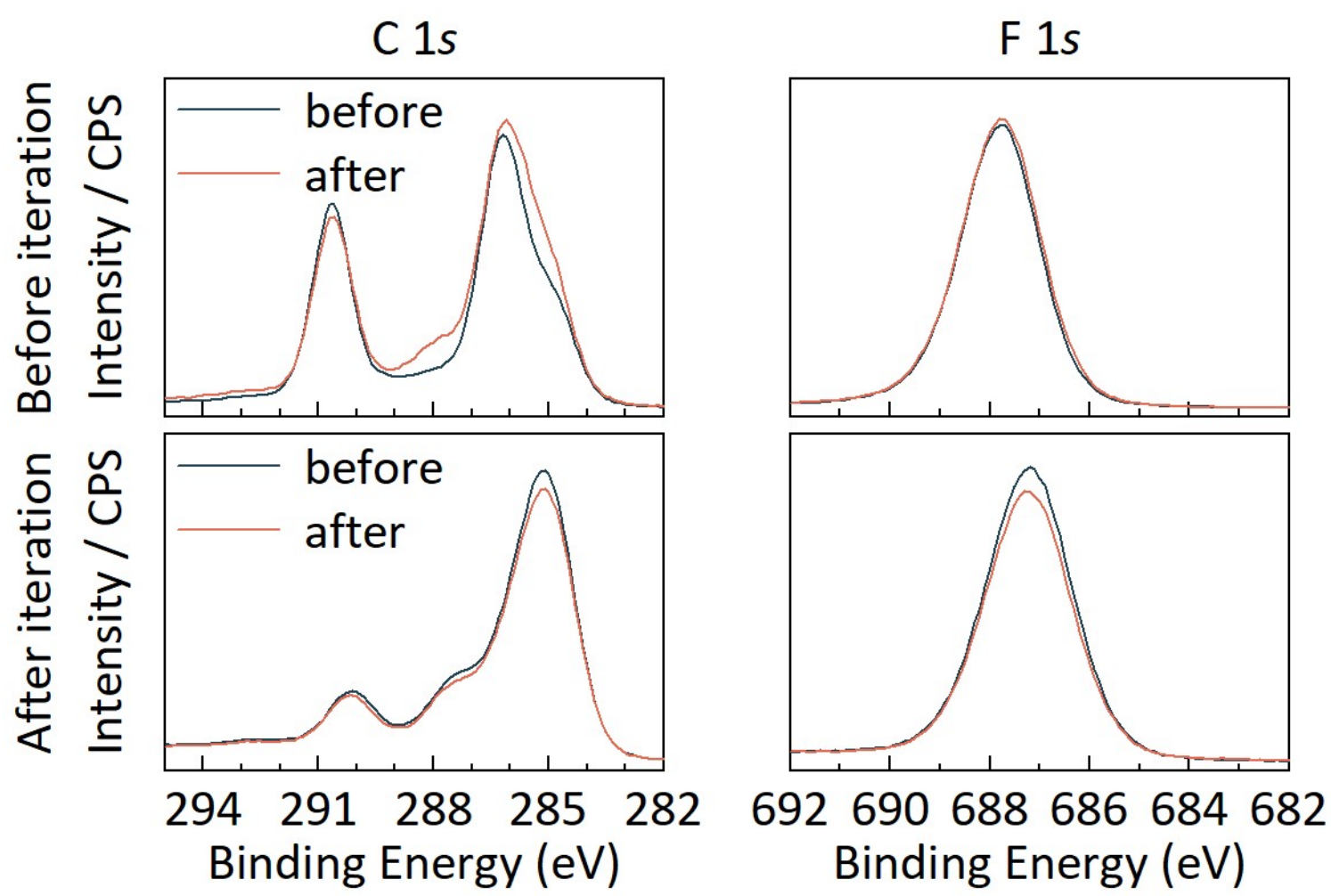

Figure S8 The change of C 1*s* and F 1*s* core state spectra for PVDF powder before and after X–ray irradiation under vacuum for 6.72 hours.

Table S1 Kinetic parameters for the global fit of the radiolysis degradation. R^2 indicates the quality of fitting.

	PVDF powder	PVDF+C65	LMFP electrode
**k_1 (h^{-1})**	0.940	1.684	0.588
**k_2 (h^{-1})**	0.058	0.073	0.037
**k_{loss} (h^{-1})**	0.058	0.091	0.071
**α**	0.544	0.315	0.222
**β**	0.595	0.454	0.287
**R^2 for C^*–F_x, F_{PVDF}**	0.9995, 0.9995	0.9971, 0.9940	0.9973, 0.9992

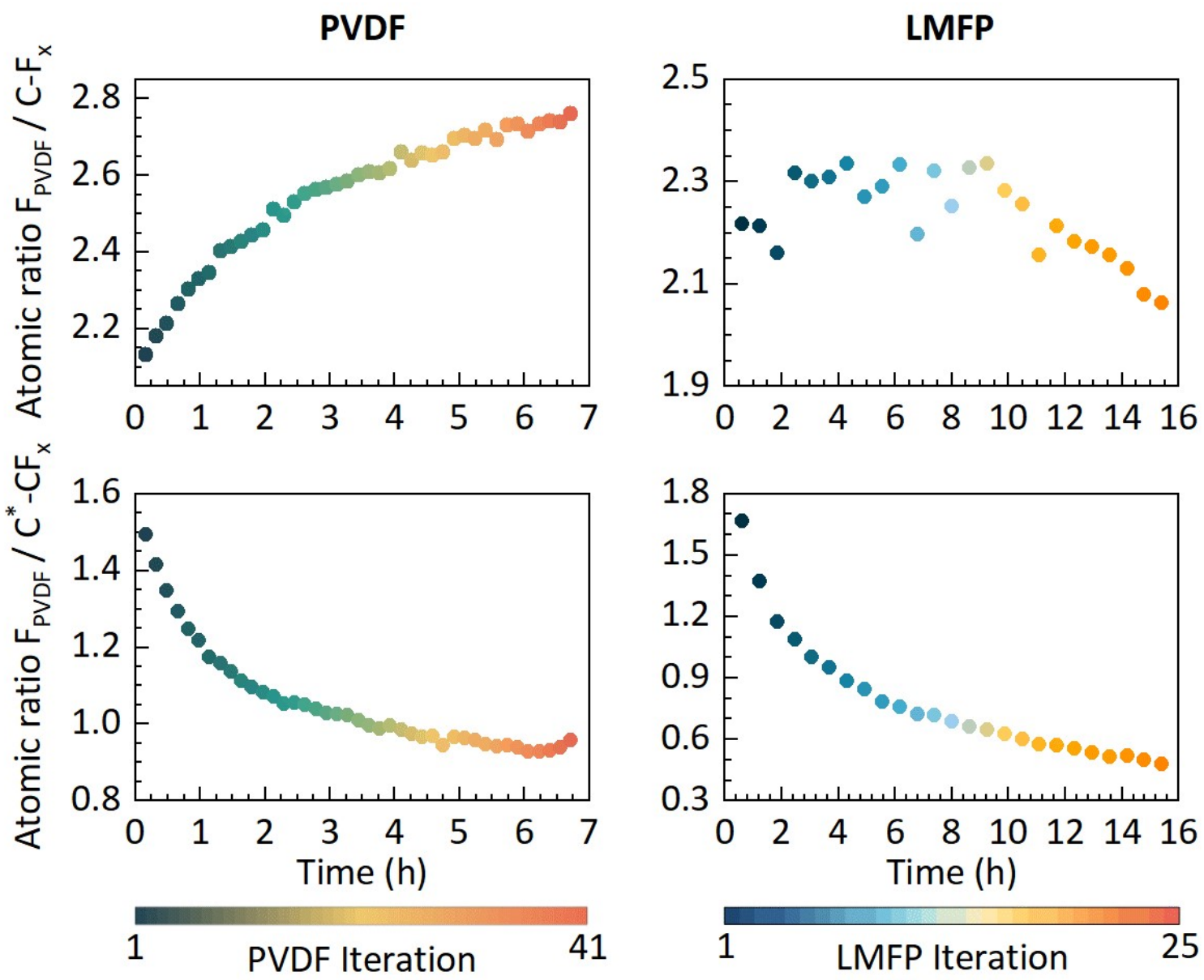

Figure S9 Temporal evolution of the $\frac{F_{PVDF}}{C-F_x}$ and $\frac{F_{PVDF}}{C^*-CF_x}$ atomic ratios of PVDF powder and LMFP electrode.

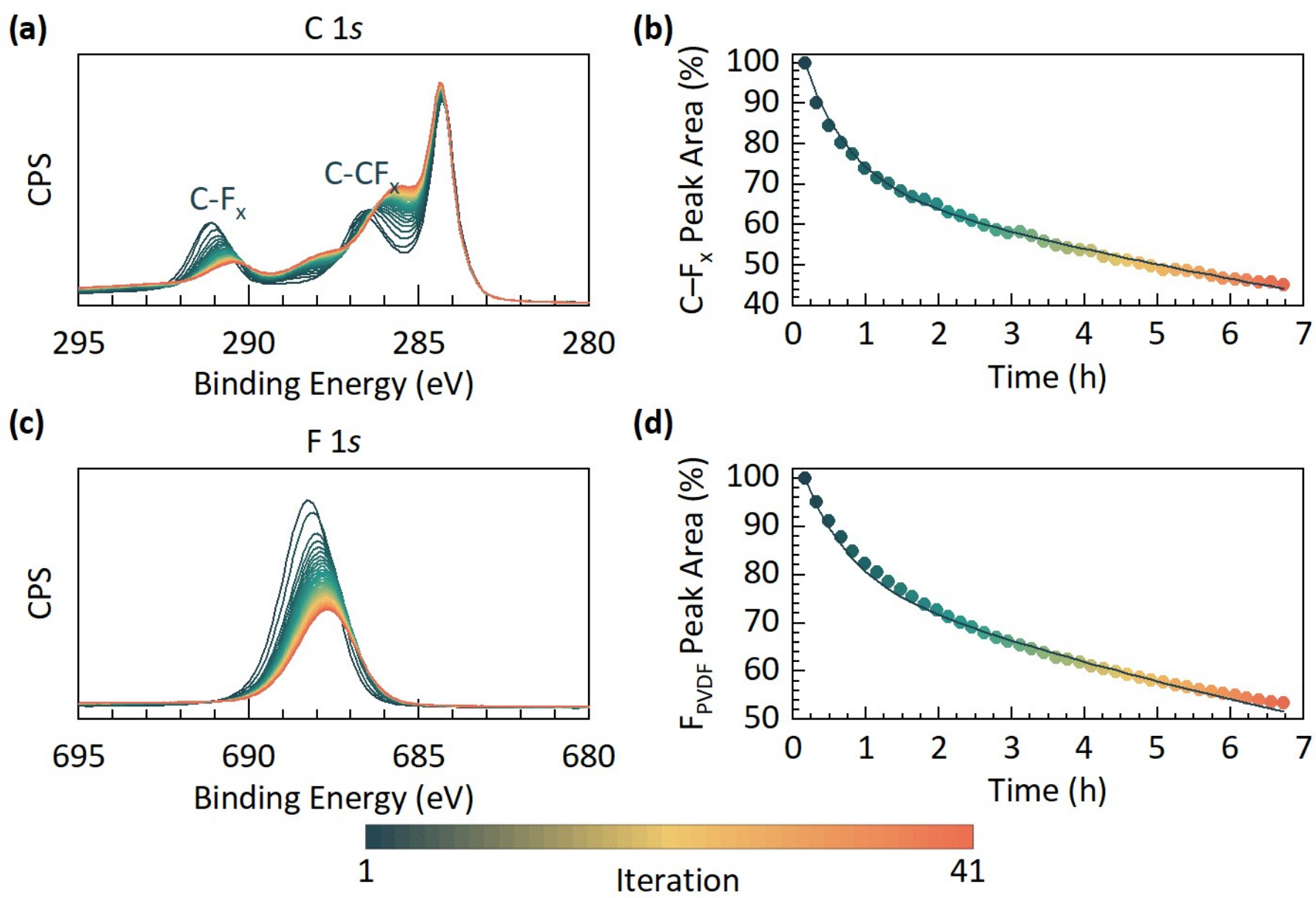

Figure S10 (a,c) XPS C 1*s* and F 1*s* core state spectral evolution of PVDF + C65 mixture under continuous, iterative scans. (b,d) Temporal evolution of C–F_x and F 1*s* peaks relative to their first iteration scans.

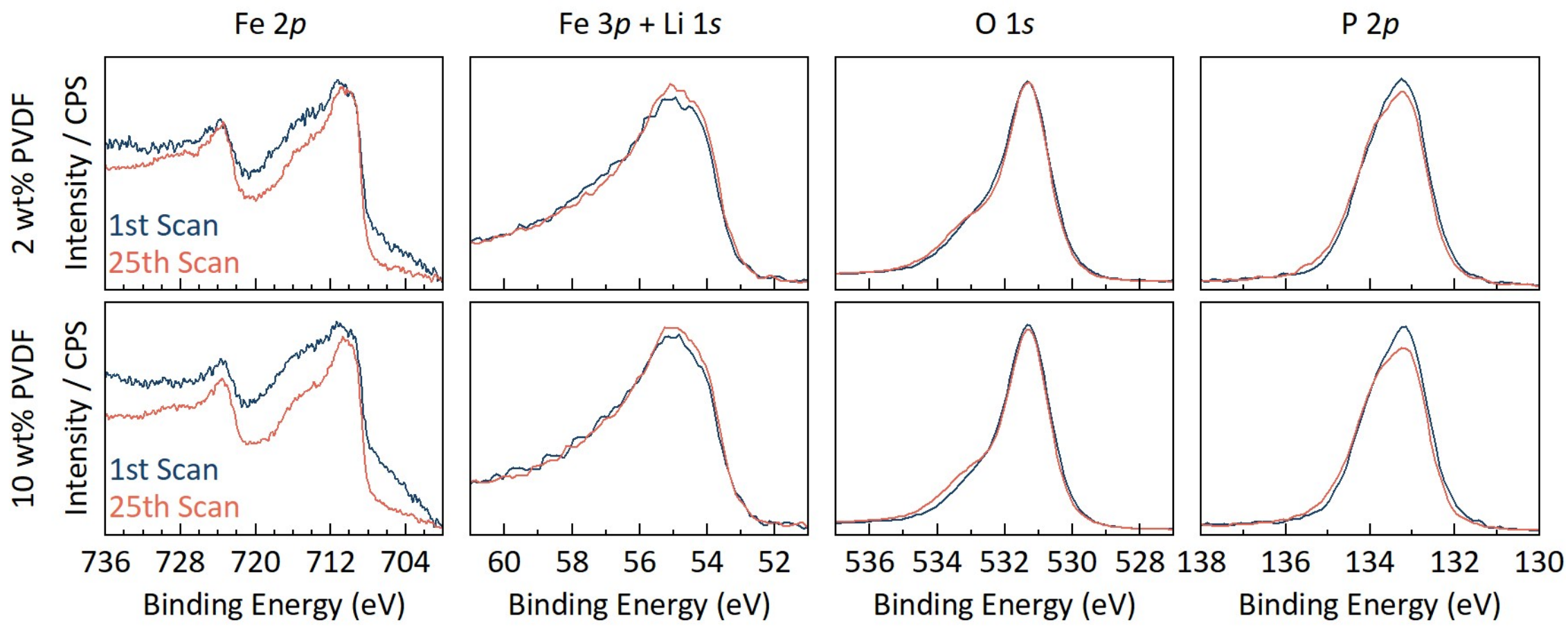

Figure S11 Li 1*s*, Fe 2*p* and 3*p*, O 1*s* and P 2*p* core state spectra of 2 wt% and 10 wt% PVDF LFP electrodes under continuous, iterative scans.

Table S2 Details of the electrode slurry formulations used in this study.

**Formulation**	**LFP mass / %**	**C45 mass / %**	**PVDF mass / %**	**Solids content / %**
1	85	5	10	45
2	93	5	2	45

Table S3 The parameters included in the RADDOSE-3D input file for X–ray dose estimation.

Crystal shape	Spherical	Beam type	Gaussian
Crystal Dimension	1 µm	Photon flux	3.80×10^{10} photons/s
PixelsPerMicron	50	FWHM	326×579 µm^2
Surrounding Elements	C2H2F2	Energy	1.487 keV
Surrounding Density	1.78 g/mL	Collimation type	Circular
		Collimation dimensions	400×710 µm^2

Table S4 Peak positions of C 1*s* of PVDF powder. FWHM of all peaks, except for the satellite peak, are constrained to be the same value in each dataset, which gradually increases from 1.20 to 1.45 upon iteration.

	PVDF peaks				Other peaks				
	C-CF_x	C-F_x	C-C	C=CF	C-C	C-O	C=O	O-C=O	Satellite
1	286.08	290.64	0	0	284.88	286.68	287.92	288.99	292.79
2	286.05	290.62	284.8	287.83	284.9	286.7	287.94	289.01	292.93
3	286	290.58	284.8	287.76	284.9	286.7	287.94	289.01	292.93
4	285.97	290.56	284.8	287.74	284.9	286.7	287.94	289.01	292.93
5	285.94	290.53	284.8	287.74	284.9	286.7	287.94	289.01	292.9
6	285.89	290.5	284.8	287.69	284.9	286.7	287.94	289.01	292.87
7	285.87	290.47	284.8	287.7	284.9	286.7	287.94	289.01	292.81
8	285.86	290.45	284.8	287.67	284.9	286.7	287.94	289.01	292.81

9	285.82	290.43	284.8	287.64	284.9	286.7	287.94	289.01	292.77
10	285.8	290.41	284.8	287.63	284.9	286.7	287.94	289.01	292.78
11	285.78	290.4	284.8	287.6	284.9	286.7	287.94	289.01	292.76
12	285.77	290.38	284.82	287.62	284.9	286.7	287.94	289.01	292.76
13	285.77	290.37	284.8	287.6	284.9	286.7	287.94	289.01	292.67
14	285.75	290.36	284.8	287.6	284.9	286.7	287.94	289.01	292.73
15	285.74	290.34	284.8	287.6	284.9	286.7	287.94	289.01	292.69
16	285.72	290.32	284.8	287.58	284.9	286.7	287.94	289.01	292.63
17	285.72	290.31	284.8	287.57	284.9	286.7	287.94	289.01	292.56
18	285.7	290.3	284.8	287.55	284.9	286.7	287.94	289.01	292.65
19	285.7	290.29	284.8	287.57	284.9	286.7	287.94	289.01	292.56
20	285.69	290.29	284.8	287.55	284.9	286.7	287.94	289.01	292.6
21	285.69	290.28	284.8	287.55	284.9	286.7	287.94	289.01	292.59
22	285.67	290.27	284.8	287.53	284.9	286.7	287.94	289.01	292.56
23	285.67	290.27	284.8	287.53	284.9	286.7	287.94	289.01	292.56
24	285.68	290.25	284.8	287.53	284.9	286.7	287.94	289.01	292.58
25	285.66	290.26	284.8	287.51	284.9	286.7	287.94	289.01	292.57
26	285.66	290.25	284.8	287.52	284.9	286.7	287.94	289.01	292.5
27	285.65	290.24	284.8	287.51	284.9	286.7	287.94	289.01	292.5
28	285.66	290.23	284.8	287.52	284.9	286.7	287.94	289.01	292.53
29	285.64	290.23	284.8	287.5	284.9	286.7	287.94	289.01	292.55
30	285.65	290.22	284.8	287.52	284.9	286.7	287.94	289.01	292.46
31	285.64	290.21	284.8	287.5	284.9	286.7	287.94	289.01	292.44
32	285.63	290.21	284.8	287.5	284.9	286.7	287.94	289.01	292.47
33	285.62	290.2	284.8	287.48	284.9	286.7	287.94	289.01	292.49
34	285.62	290.2	284.8	287.48	284.9	286.7	287.94	289.01	292.45
35	285.63	290.19	284.8	287.48	284.9	286.7	287.94	289.01	292.41
36	285.62	290.19	284.8	287.49	284.9	286.7	287.94	289.01	292.48
37	285.62	290.18	284.8	287.48	284.9	286.7	287.94	289.01	292.45
38	285.61	290.18	284.8	287.48	284.9	286.7	287.94	289.01	292.43
39	285.6	290.17	284.8	287.46	284.9	286.7	287.94	289.01	292.41
40	285.61	290.16	284.78	287.44	284.9	286.7	287.94	289.01	292.45
41	285.61	290.16	284.8	287.45	284.9	286.7	287.94	289.01	292.4

Table S5 Peak areas of C 1*s* of PVDF powder. The peak areas of oxygen peaks in 'carbon tape' are allowed to reduce during the initial stage to align with the area decrease in O 1*s*. FWHM of all peaks, except for the satellite peak, are constrained to be the same value in each dataset, which gradually increases from 1.20 to 1.45 upon iteration.

	PVDF peaks				Carbon tape peaks				
	C-CF_x	C-F_x	C-C	C=CF	C-C	C-O	C=O	O-C=O	Satellite
1	31165.21	21863.26	0	0	11388.96	5508.13	4036.6	1716.45	684.86
2	31879.45	20690.39	840.35	1273.75	11403	5270.13	3862.17	1642.16	810.35
3	32452.71	19767.88	1747.98	2153.68	11403	5164.36	3784.66	1609.2	906.12
4	32976.05	18850.21	2606.26	3028.01	11403	4948.12	3626.19	1541.82	900.14
5	33548.88	18174.8	3492.96	3749.87	11403	4770.37	3495.93	1486.44	1026.93
6	33573.34	17566.1	4299.48	4192.22	11403	4793.56	3512.94	1493.67	1239.06

7	34090.98	17077.17	5238.12	4792.39	11403	4538.1	3325.71	1414.06	1251.16
8	34043.21	16428.57	5997.57	5150.24	11403	4390.47	3217.53	1368.06	1347.3
9	34076.12	16036.3	6743.14	5447.05	11403	4390.47	3217.53	1368.06	1397.44
10	34237.36	15675.99	7263.08	5634.94	11403	4390.47	3217.53	1368.06	1444.39
11	34232.03	15343.77	7750.77	5940.46	11403	4390.47	3217.53	1368.06	1402.48
12	34225.21	15094.47	8687.88	5970.8	11403	4390.47	3217.53	1368.06	1475.69
13	34121.95	14553.05	9355.19	6246.19	11403	4390.47	3217.53	1368.06	1606.5
14	34291.46	14483.46	9723.86	6386.03	11403	4390.47	3217.53	1368.06	1687.29
15	33773.87	14091.74	10786.19	6374.78	11403	4390.47	3217.53	1368.06	1780.02
16	33630.83	13841.65	11414.09	6551.27	11403	4390.47	3217.53	1368.06	1806.11
17	33553.92	13594.01	12135.61	6712.96	11403	4390.47	3217.53	1368.06	1812.02
18	33567.24	13459.67	12284.77	6821.9	11403	4390.47	3217.53	1368.06	1919.12
19	33297.31	13263.4	13201.76	6799.1	11403	4390.47	3217.53	1368.06	1977.89
20	33086.98	13079.11	13612.59	6987.35	11403	4390.47	3217.53	1368.06	1947.23
21	33183.2	12868.24	14118.67	7020.47	11403	4390.47	3217.53	1368.06	1939.72
22	33417.46	12742.83	14116.57	7209.46	11403	4390.47	3217.53	1368.06	1905.87
23	33384.65	12643.88	14410.88	7166.03	11403	4390.47	3217.53	1368.06	2065.73
24	32790.14	12465.57	15423.21	7249.27	11403	4390.47	3217.53	1368.06	2182.32
25	32767.33	12137.89	15515.14	7379.81	11403	4390.47	3217.53	1368.06	1884.77
26	32999.46	12169.2	15779.63	7386.33	11403	4390.47	3217.53	1368.06	2056.35
27	32887.08	11963.31	16299.43	7528.76	11403	4390.47	3217.53	1368.06	2157.5
28	32506.68	11886.68	16893.89	7336.46	11403	4390.47	3217.53	1368.06	2099.76
29	33107.46	11769.03	16700.04	7574	11403	4390.47	3217.53	1368.06	2073.17
30	32204.73	11546.56	17772.68	7466.65	11403	4390.47	3217.53	1368.06	2088.26
31	32135.68	11434.68	18032.9	7575.85	11403	4390.47	3217.53	1368.06	2184.15
32	32044.16	11377.54	18212.07	7807.17	11403	4390.47	3217.53	1368.06	2271.2
33	32067.49	11186.27	18591.49	7757.91	11403	4390.47	3217.53	1368.06	2243.45
34	32142.38	11232.64	18906.08	7833.82	11403	4390.47	3217.53	1368.06	2253.18
35	31746.95	10977.16	19312.6	7695.26	11403	4390.47	3217.53	1368.06	2131.48
36	31819.2	10925.32	19643.74	7789.81	11403	4390.47	3217.53	1368.06	2266
37	31843.26	10899.71	20145.29	7900.99	11403	4390.47	3217.53	1368.06	2283.6
38	31792.09	10783.11	20158.48	7950.8	11403	4390.47	3217.53	1368.06	2382.28
39	31455.42	10684.68	20892.45	7967.51	11403	4390.47	3217.53	1368.06	2252.05
40	31004.45	10622.3	21695.05	8002.01	11403	4390.47	3217.53	1368.06	2323.17
41	30262.44	10502.14	22510.57	7937.16	11403	4390.47	3217.53	1368.06	2208.31
